# Global transcriptional response of *Escherichia coli *O157:H7 to growth transitions in glucose minimal medium

**DOI:** 10.1186/1471-2180-7-97

**Published:** 2007-10-29

**Authors:** Teresa M Bergholz, Lukas M Wick, Weihong Qi, James T Riordan, Lindsey M Ouellette, Thomas S Whittam

**Affiliations:** 1 Microbial Evolution Laboratory, National Food Safety and Toxicology Center, Michigan State University, East Lansing, Michigan 48824, USA

## Abstract

**Background:**

Global patterns of gene expression of *Escherichia coli *K-12 during growth transitions have been deeply investigated, however, comparable studies of *E. coli *O157:H7 have not been explored, particularly with respect to factors regulating virulence genes and genomic islands specific to this pathogen. To examine the impact of growth phase on the dynamics of the transcriptome, O157:H7 Sakai strain was cultured in MOPS minimal media (0.1% glucose), RNA harvested at 10 time points from early exponential to full stationary phase, and relative gene expression was measured by co-hybridization on high-density DNA microarrays. Expression levels of 14 genes, including those encoding Shiga toxins and other virulence factors associated with the locus of enterocyte effacement (LEE), were confirmed by Q-PCR.

**Results:**

Analysis of variance (R/MAANOVA, Fs test) identified 442 (36%) of 1239 O157-specific ORFs and 2110 (59%) of 3647 backbone ORFs that changed in expression significantly over time. QT cluster analysis placed 2468 of the 2552 significant ORFs into 12 groups; each group representing a distinct expression pattern. ORFs from the largest cluster (*n *= 1078) decreased in expression from late exponential to early stationary phase: most of these ORFs are involved in functions associated with steady state growth. Also represented in this cluster are ORFs of the TAI island, encoding tellurite resistance and urease activity, which decreased ~4-fold. Most ORFs of the LEE pathogenicity island also decreased ~2-fold by early stationary phase. The ORFs encoding proteins secreted via the LEE encoded type III secretion system, such as *tccP *and *espJ*, also decreased in expression from exponential to stationary phase. Three of the clusters (*n *= 154) comprised genes that are transiently upregulated at the transition into stationary phase and included genes involved in nutrient scavenging. Upregulated genes with an increase in mRNA levels from late exponential to early stationary phase belonged to one cluster (*n *= 923) which includes genes involved in stress responses (e.g. *gadAB*, *osmBC*, and *dps*). These transcript levels remained relatively high for > 3 h in stationary phase. The Shiga toxin genes (*stx*1AB and *stx*2B) were significantly induced after transition into stationary phase.

**Conclusion:**

Expression of more than 300 O157-specific ORFs, many implicated in virulence of the O157 pathogen, was modulated in a growth dependent manner. These results provide a baseline transcriptional profile that can be compared to patterns of gene expression of this important foodborne pathogen under adverse environmental conditions.

## Background

Enterohemorrhagic *Escherichia coli *(EHEC), a food and water- borne pathogen of zoonotic origin, are an important cause of acute gastroenteritis in humans. O157:H7 is the predominant serotype of EHEC causing illness in the United States [1]. One of the hallmarks of EHEC pathogenesis is the formation of attaching and effacing lesions on intestinal epithelial cells [2,3], resulting in intimate adherence of the bacterial cell to the intestinal mucosa. The ability to form A/E lesions is encoded by the Locus of Enterocyte Effacement (LEE), a 35 kb pathogenicity island [4]. Another principal virulence characteristic of EHEC is the production of Shiga toxins, two component cytotoxins that inhibits protein synthesis in eukaryotic cells [5]. The genes for production of Shiga toxin (*stx1 *and *stx2*) are located in intact or partial genomes of lambda prophages that are inserted into the chromosome [6,7]. Together, the LEE and Shiga toxins are considered to be two of the principal virulence determinants that mediate development of hemorrhagic colitis and the life-threatening hemolytic uremic syndrome [8].

Regulation of these virulence factors is influenced by global regulators as well as regulators specific to the virulence factor. Many of the virulence factors in EHEC are carried on 'foreign' DNA, DNA which is not present in *E. coli *K-12, and their expression is typically regulated by transcriptional regulators also carried on these mobile elements. EHEC can utilize global regulators that are common to all *E. coli *as well as O157-specific regulators to control expression of virulence factors [8,9]. For example, the expression of LEE is dependent on growth phase and responds to nutrient downshifts via ppGpp signaling through DksA to Ler (LEE encoded regulator) and PchA [10]. Also, LEE expression can be upregulated by activation of Ler in the presence of bicarbonate ions [11], and by quorum sensing, in which autoinducer 3 activates LEE expression through Ler [12]. Shiga toxin expression is linked to expression of the phage lysis genes [13,14] and can be controlled by the iron responsive regulator Fur [15]. In addition to the known virulence determinants, *E. coli *O157:H7 Sakai and EDL-933 contain ~1.3 Mb of sequence not found in *E. coli *K-12; these sequences occur as part of the genetic material of 18 lysogenic phages and 6 phage-like elements that have integrated into the Sakai genome [16] and as 177 O157 strain-specific islands (OI number) in the EDL-933 strain [17]. Recently it was identified that many effector proteins that are secreted via the LEE-encoded type three secretion system are specified by genes distributed throughout the genome on lambdoid phages [18].

In general, *E. coli *populations are ecologically and metabolically versatile and can adapt to growth under a wide range of conditions, adaptations essential to a bimodal lifestyle either in the primary habitat within animal hosts or in secondary habitats as free living cells in the natural environment [19]. Slowing metabolic activity during stationary phase reflects a survival mechanism in nutrient poor environments in which bacteria undergo a variety of morphological and physiological changes. In stationary phase, *E. coli *are more resistant to a number of stresses, including pH stress [20,21] and osmotic stress [22], both of which occur during transit through the colonization of the host intestinal tract. Important to the transmission route of *E. coli *O157:H7 is the ability to persist in adverse environments until entering a new host. Hence studying growth transitions and how they relate to the lifestyle of *E. coli *O157:H7 is essential to understanding the persistence and spread of this pathogen from the bovine reservoir to foods and to humans.

Microarrays have been utilized to observe changes induced in the transcriptome of *E. coli *during growth transitions and in response to different environmental stresses. Global transcription profiling of *E. coli *K-12 indicates a coordinated response to growth arrest [23]. The sigma factor RpoS plays a crucial role in transcription of stationary phase and stress response genes [24-26]. Microarray studies have been conducted to understand how *E. coli *K-12 responds to growth at low pH [27,28], on rich and minimal media [29], during anaerobic growth [30-32], and growth transitions in rich medium [33]. Transcriptional responses of O157:H7 during growth on plasma membranes [34] and upon exposure to norfloxacin [35], have been investigated, and microarrays have been used to identify genes regulated by *luxS *in *E. coli *O157:H7 [36], but a full picture of global gene expression during growth transitions, although well-studied in *E. coli *K-12, has not been investigated in the food borne pathogen *E. coli *O157:H7.

A key component to understanding the adaptations and ecology of *E. coli *O157:H7 is to determine how the O157-specific genomic elements respond to one of the critical stages of growth, stationary phase. It has been documented that some virulence factors are regulated by environmental cues that signal the presence of the host environment, but what is less understood is how these virulence factors are expressed in a non-host environment. Here we address the question of how O157:H7 responds to growth transitions by determining the changes in global gene expression patterns in O157:H7 during the transition from exponential to stationary phase in minimal medium, with particular interest in expression patterns of O157-specific genes. The dynamics of the transcriptome of *E. coli *O157:H7 Sakai was investigated by comparative microarray hybridizations at 10 different time points during exponential and stationary phase and compared to obtain a temporal pattern of gene expression. Gene expression patterns were also verified by Q-PCR of 14 ORFs including those encoding principal virulence factors (e.g. LEE-encoded proteins and Shiga toxins).

## Results

### Analysis of expression ratios

**Figure 1:**
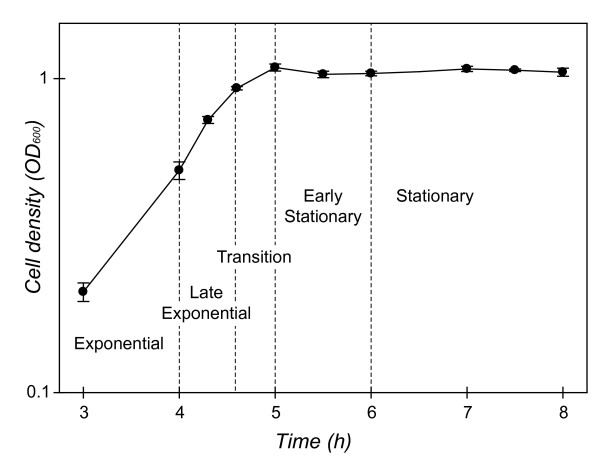
**Average growth of *E. coli *O157:H7 Sakai in MOPS minimal medium**. Increase in cell density is measured at OD_600 _at 10 time periods (hr) of growth into stationary phase. Error bars represent the standard deviation of four culture replicates. Samples were taken from the culture at the time points plotted and RNA was extracted. The 5 growth phases, separated by dotted lines, are defined as exponential growth, late exponential growth, transition to stationary phase, early stationary phase, and stationary phase.

**Figure 2:**
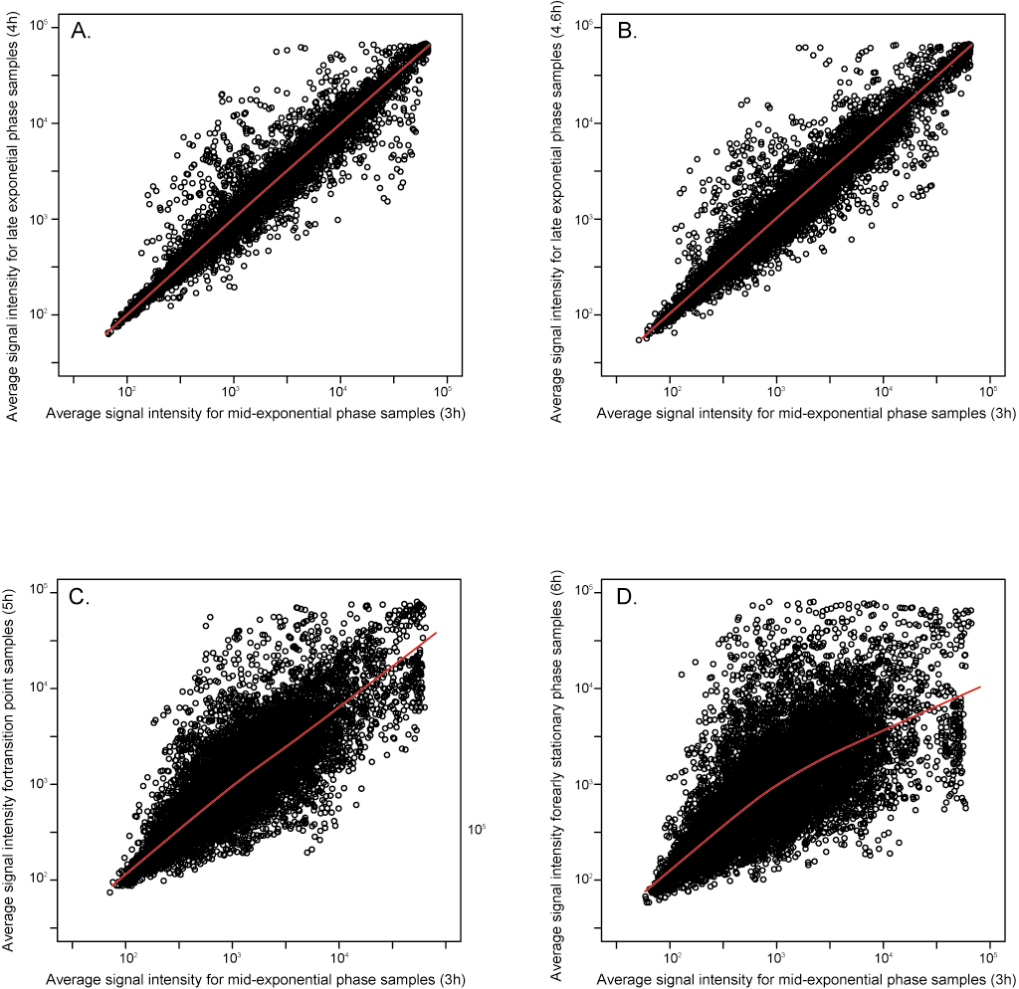
**Average signal intensity plots for four of the time points of growth**. The normalized signals of 4,886 ORFs for *E. coli *O157:H7 Sakai were averaged from four hybridizations, representing four biological replicates: **A**. Average signal intensity for exponential phase (3 h) vs late exponential phase (4 h). **B**. Average signal intensity for exponential phase (3 h) vs late exponential phase (4.6 h). **C**. Average signal intensity for exponential phase (3 h) vs transition to stationary phase (5 h). **D**. Average signal intensity for exponential phase (3 h) vs. stationary phase (6 h. The Lowess line is plotted for each graph in red.

The results are based on O157:H7 Sakai cultures collected from 10 time points of growth in minimal media with 4 replicates (i.e. 4 independent cultures that were each sampled 10 times) for a total of 40 samples. The resulting array data have been deposited in the NCBI Gene Expression Omnibus, accession GSE7477. The samples collected from the mid-exponential phase 3-hour time point (Fig. 1) were used as a common reference for hybridization and analysis for all subsequent time points in a replicated reference design where all the samples are biologically replicated, including the reference [37]. This design allowed for indirect comparison between all time points via the 3-hour samples [37,38]. We incorporated the dye-swaps among the four biological replicates which can confound dye effects and biological replicate effects. However, if there is significant variation in the rate of dye incorporation from one labeling reaction to another, this would result in large dye-effects compared to biological replicate effect [39], an effect not found in our experiments. In general, the variance estimates for the biological replicate effects were small (median 0.0008), compared to the variance estimates (<0.5%) for the array effects (median 0.16). Most ORFs (4362/5886, 90%) had similar signal intensities for the mid- and late- exponential phase samples, indicating that there were few overall differences in expression between these time points (Fig. 2A and 2B). In contrast, there were many differences in expression found between mid-exponential phase samples and samples from the transition point (Fig. 2C) and early stationary phase (Fig. 2D).

### Summary of significant changes in gene expression over time

Significant changes in expression through time were determined using ANOVA and the Fs-test [40] for the 4,886 O157:H7 Sakai ORFs targeted on the microarray. ANOVA does not capture how time affects gene expression, but how expression changes over time. The *p *values were adjusted to correct for type I error using the Benjamini-Hochberg (B-H) false discovery rate approach with the linear step-up correction implemented in R/MAANOVA. The B-H correction for multiple testing applies a step-wise correction to the *p *values, and allows selection of a suitable *p *value cutoff based on practical considerations. The 2,552 ORFs with the lowest adjusted *p *values (< 0.0000001) were considered for further analysis (Additional File 1). Fold change in expression between time points was determined by calculating the difference in log_2 _expression between the time points of interest (Additional Files 1 and 2).

### Trends in gene expression identified by significant contrasts between subsequent time points and QT clustering

**Figure 3:**
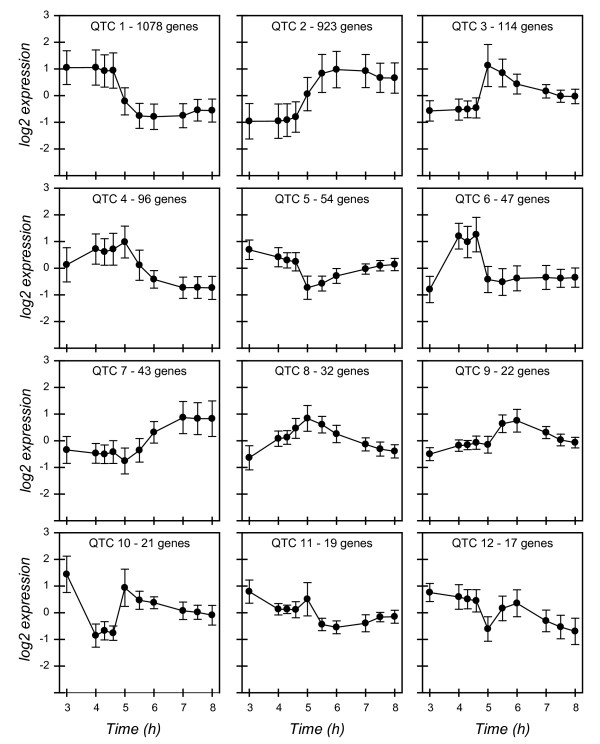
**Quality Threshold (QT) clusters of the 2,552 ORFs with significant changes in gene expression**. The number of ORFs in each cluster is listed at the top of each plot. Average expression profiles were determined for the ORFs in each cluster and plotted with the standard deviation for each time point.

Expression profiles of the 2,552 ORFs with significant differences were clustered into 12 groups using Quality Threshold (QT) clustering (Fig. 3), which groups similar expression profiles based on jackknife correlations [41]. The majority of ORFs were classified into 12 groups; only 84 were not placed into a cluster. ORFs were also classified based on the time intervals at which a significant change in gene expression occurred. A subset of 524 ORFs had differences in expression from mid- to late-exponential phase (3 h to 4 h). The majority of ORFs, 1,668 of 4,886 (34%), had significantly changed in transcript level at the stationary phase transition point (4.6 to 5 h). Additionally, 1,045 of 4,886 (20%) ORFs significantly changed during the transition point to early stationary phase (5 to 5.5 h).

### Significant changes in exponential phase – response to limiting oxygen

**Figure 4:**
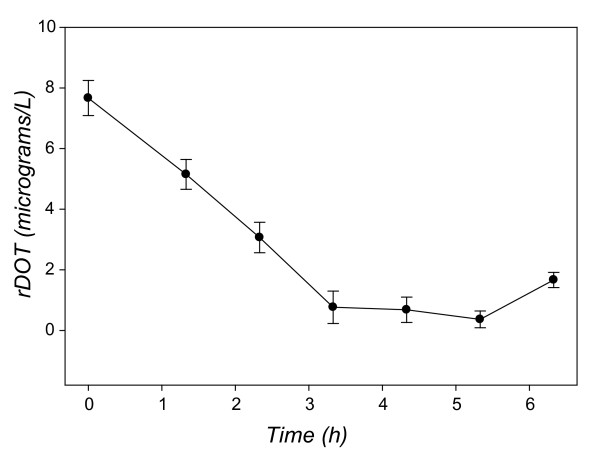
**Residual dissolved oxygen (O_2_) in MOPS minimal medium**. The mean rDOT was determined for three independent cultures of *E. coli *O157:H7 Sakai in glucose limited MOPS minimal medium and plotted with the error bars indicating the standard deviation.

There were 524 ORFs with significant differences in transcript level during exponential phase – a total of 113 genes changed only in exponential phase, whereas the remainder had altered levels of expression in exponential as well as stationary phase. ORFs that decreased in expression included those encoding enzymes of the TCA cycle, such as *sucA*, and *sdhABCD *(Table 1, Fig. 3; QTC 10). Many of the ORFs (94/524, 18%) that changed in transcript level are regulated by the transcription factor FNR [31,32], whereas a smaller set of ORFs (29/524, 5%) are known to be regulated by ArcA [42]. Genes that increased included those encoding anaerobic electron acceptors, fumarate reductase, *frdAB*, and DMSO reductase, *dmsA *and *dmsC*, as well as a DMSO reductase paralog, *ynfE *(Table 1, Fig. 3; QTC 6). Genes encoding the cytochrome c biogenesis system (*ccmABCDEFG) *and the cytochrome c-like protein (*napB *and *napC*) also had a significant increase from 3 h to 4 h. The operon encoding the low affinity cytochrome oxidase *bo *(*cyoABCD*) decreased 9 to15- fold in expression from 3 h to 4 h (Table 1). Concomitantly, expression of the high affinity cytochrome oxidase *bd*, *cydAB*, increased 4–5 fold. Expression of the high and low affinity cytochrome oxidases is directly influenced by the level of available oxygen in the media [43,44]. Gene set enrichment analysis (GSEA) found that genes involved in anaerobic energy metabolism were significantly enriched in the 4 h sample compared to the 3 h sample, and that genes involved in aerobic energy metabolism and the TCA cycle were significantly enriched in the 3 h compared to the 4 h culture (Additional File 3). The altered expression of the cytochrome oxidases as well as the increase in FNR-controlled, anaerobic associated genes and decrease in TCA cycle enzymes is consistent with the hypothesis that oxygen became limiting under the culture conditions that were used. Subsequent measurement of the residual dissolved oxygen tension in the culture during growth indicates that residual dissolved O_2 _levels decreased almost to zero (95% decrease in O_2_) during exponential phase (Fig. 4). As cultures entered stationary phase, the O_2 _levels began to increase, as the demand for O_2 _by the culture decreased.

**Table 1:** Significant ORFs (*p *value < 1 × 10^7^) with greater than 4-fold significant change between 3 h and 4 h.

Ecs number	Gene^a^	function	log2 change in expression	QT cluster^b^
ECs0236	O157	Unknown function	-2.89	10
ECs0482	***cyoE***	protoheme IX farnesyltransferase	-2.08	10
ECs0483	***cyoD***	cytochrome o ubiquinol oxidase subunit IV	-3.18	10
ECs0484	***cyoC***	cytochrome o ubiquinol oxidase subunit III	-3.32	10
ECs0485	***cyoB***	cytochrome o ubiquinol oxidase subunit I	-3.87	10
ECs0486	***cyoA***	cytochrome o ubiquinol oxidase subunit II	-3.89	10
ECs0660	***dcuC***	transport of dicarboxylates	2.20	6
ECs0746	*sdhC*	succinate dehydrogenase, cytochrome b556	-3.15	10
ECs0747	*sdhD*	succinate dehydrogenase, hydrophobic subunit	-2.66	10
ECs0748	*sdhA*	succinate dehydrogenase	-3.06	10
ECs0749	*sdhB*	succinate dehydrogenase	-2.79	10
ECs0750	*-*	orf, hypothetical protein	-2.24	10
ECs0751	*sucA*	2-oxoglutarate dehydrogenase	-2.20	10
ECs0768	***cydA***	cytochrome d terminal oxidase	2.56	4
ECs0769	***cydB***	cytochrome d terminal oxidase	2.58	4
ECs0916	*yliH*	putative receptor	3.84	2
ECs0979	***dmsA***	anaerobic dimethyl sulfoxide reductase	3.71	4
ECs0981	***dmsC***	anaerobic dimethyl sulfoxide reductase	2.08	4
ECs0986	***pflB***	formate acetyltransferase 1	2.04	6
ECs1572	***pepT***	putative peptidase T	2.64	2
ECs1728	***narK***	nitrite extrusion protein	2.98	6
ECs1729	***narG***	nitrate reductase 1, alpha subunit	2.25	4
ECs1741	***adhE***	CoA-linked acetaldehyde dehydrogenase	2.50	6
ECs1756	***yciD***	putative outer membrane protein	4.15	4
ECs1875	*ycjL*	probable amidotransferase subunit	-2.28	2
ECs2027	*ydcI*	putative transcriptional regulator LYSR-type	-2.41	2
ECs2078	***fdnG***	formate dehydrogenase-N, nitrate-inducible	2.30	6
ECs2150	***ydfZ***	orf, hypothetical protein	4.70	4
ECs2293	***ynfE***	putative oxidoreductase, major subunit	2.83	6
ECs2457	***ydjY***	orf, hypothetical protein	2.03	6
ECs2463	***ynjE***	putative thiosulfate sulfur transferase	3.47	1
ECs2614	*yecH*	orf, hypothetical protein	3.09	6
ECs3061	*fruB*	PTS system, fructose-specific IIA/fpr component	2.02	1
ECs3084	*dsbE*	disulfide oxidoreductase	2.04	1
ECs3088	*ccmC*	heme exporter protein C	2.19	1
ECs3090	*ccmA*	ATP binding protein of heme exporter A	2.64	1
ECs3091	***napC***	cytochrome c-type protein	3.55	1
ECs3092	***napB***	cytochrome c-type protein	3.30	1
ECs3093	***napH***	ferredoxin-type protein: electron transfer	3.04	un
ECs3180	***ackA***	acetate kinase	2.28	6
ECs3226	*yfcZ*	orf, hypothetical protein	3.02	6
ECs3445	***yfiD***	putative formate acetyltransferase	4.48	6
ECs3460	*yfiA*	putative yhbH sigma 54 modulator	2.12	2
ECs3582	***hypA***	pleiotrophic effects on 3 hydrogenase isozymes	2.92	6
ECs3583	***hypB***	guanine-nucleotide binding protein	2.10	6
ECs3586	***hypE***	plays structural role in maturation of all 3 hydrogenases	2.10	6
ECs3799	O157	orf; hypothetical protein	2.92	6
ECs3800	O157	orf; hypothetical protein	2.33	6
ECs3802	O157	putative ATP-binding protein of ABC transport system	2.40	6
ECs3833	***ansB***	periplasmic L-asparaginase II	3.41	4
ECs3880	*hybB*	probable cytochrome Ni/Fe component of hydrogenase-2	2.05	4
ECs4040	*yhbV*	orf, hypothetical protein	3.11	1
ECs4216	***nirB***	nitrite reductase (NAD(P)H) subunit	3.55	1
ECs4343	***nikA***	periplasmic binding protein for nickel	3.46	1
ECs4344	***nikB***	transport of nickel, membrane protein	2.10	6
ECs4347	***nikE***	ATP-binding protein of nickel transport system	2.85	1
ECs4398	***yhjA***	putative cytochrome C peroxidase	2.86	6
ECs4456	*yiaI*	orf, hypothetical protein	2.40	4
ECs4750	*yigI*	orf, hypothetical protein	-2.55	10
ECs4834	***sodA***	superoxide dismutase, manganese	-2.40	7
ECs4874	*gldA*	glycerol dehydrogenase, (NAD)	2.86	6
ECs5052	***nrfA***	periplasmic cytochrome c(552)	3.08	un
ECs5053	***nrfB***	formate-dependent nitrite reductase	2.86	6
ECs5054	***nrfC***	formate-dependent nitrite reductase; Fe-S centers	2.96	un
ECs5134	***frdB***	fumarate reductase, anaerobic, iron-sulfur protein subunit	2.50	8
ECs5135	***frdA***	fumarate reductase, anaerobic, flavoprotein subunit	2.11	8
ECs5214	***nrdG***	anaerobic ribonucleotide reductase activating protein	2.02	6
ECs5215	***nrdD***	anaerobic ribonucleoside-triphosphate reductase	3.54	6
ECs5298	*yjiM*	orf, hypothetical protein	2.76	8

^a^Genes in bold have been identified as being under the control of the transcriptional regulator FNR [29, 30]. Underlined genes have been identified as being controlled by the transcriptional regulator ArcA [40].

^b^QT cluster un. means unassigned

### Significant change in O157-specific genes during exponential phase

In contrast to the backbone genes, only a small proportion (6%) of the O157-specific ORFs changed significantly in expression measured during exponential phase. While statistically significant, most of the differences in expression level for these 61 genes were not substantial, ranging from 1.2- to 2- fold (Additional File 2). Many of these ORFs are of unknown function, but of the 45 O157-specific ORFs that increased from 3–4 h, 9 of them were from a single Sakai phage (Sp) 15 region which encodes the Shiga toxin 1 phage, and contains 5 ORFs from Sp 5 which encodes the Shiga toxin 2 phage. Interestingly, ECs2381 a locus whose expression has been shown to contribute to colonization in the bovine gastrointestinal tract [45], was also significantly induced 1.8 fold from 3 to 4 h. There were 4 additional O157-specific ORFs that had a greater than 4-fold change in expression from 3–4 h (Table 1). These 4 ORFs were not associated with any of the Sakai phages, and have unknown or putative functions.

### Genes with significant changes in expression at the stationary phase transition point

The majority of significant changes in transcript level occurred at the stationary phase transition point. There were 176 ORFs with a 4-fold or greater change in expression at the stationary phase transition point (Table 2). A total of 363 ORFs that significantly increased or decreased during the transition to stationary phase (4.6–5 h) also increased or decreased in early stationary phase (5–5.5 h) (Table 3). A reduced level of expression of ribosomal genes and genes involved in nucleotide and amino acid synthesis was observed from 4.6–5 h and 5–5.5 h (Tables 2 and 3, Fig. 3; QTC 1). Many of the genes that were significantly down-regulated upon entry into stationary phase have been also observed in *E. coli *K-12 [23], including decreased expression of the molecular chaperones encoded by *groEL, htpG*, and *dnaK *(Tables 2 and 3, Fig. 3; QTC 1, Additional File 1).

**Table 2:** Significant ORFs (p value < 1 × 10^7^) with greater than 4-fold significant changes in expression at the stationary phase transition point (4.6 to 5 h).

Ecs no.	Gene	log2 change in expression	QT cluster	Ecs no.	Gene	log2 change in expression	QT cluster
ECs0034	*dapB*	-2.63	1	ECs1286	O157	-2.09	1
ECs0035	*carA*	-4.23	1	ECs1426	*mdoG*	-2.46	5
ECs0068	*araC*	2.57	2	ECs1440	*pyrC*	-2.65	1
ECs0072	*tbpA*	-2.25	1	ECs1468	*plsX*	-2.16	1
ECs0083	*fruL*	-2.06	1	ECs1479	*ptsG*	-3.09	1
ECs0124	*speD*	-2.74	1	ECs1492	*mfd*	-2.33	1
ECs0149	*dksA*	2.12	2	ECs1604	*ycfC*	-2.19	1
ECs0150	*sfsA*	2.83	2	ECs1662	O157	-2.53	1
ECs0191	*yaeO*	2.42	2	ECs1684	*dadA*	2.34	2
ECs0207	*dniR*	-2.11	1	ECs1710	*ychH*	4.61	2
ECs0334	O157	-2.07	1	ECs1712	*prsA*	-2.68	1
ECs0374	O157	2.42	2	ECs1859	*rnb*	-2.70	1
ECs0401	*mhpR*	4.33	3	ECs1954	O157	-2.61	1
ECs0416	O157	3.55	2	ECs1955	O157	-3.22	1
ECs0468	*ribH*	-2.27	1	ECs2011	*ynbB*	2.34	2
ECs0483	*cyoD*	2.06	10	ECs2021	*aldA*	3.60	2
ECs0485	*cyoB*	2.01	10	ECs2027	*ydcI*	3.35	2
ECs0489	*bolA*	2.10	2	ECs2031	*ydcH*	2.99	2
ECs0584	*purK*	-2.27	1	ECs2037	*ydcN*	2.71	2
ECs0585	*purE*	-2.66	1	ECs2083	*sfcA*	-2.18	1
ECs0610	*cusC*	-2.31	1	ECs2182	O157	3.21	2
ECs0611	*cusF*	-2.82	1	ECs2293	*ynfE*	-2.97	6
ECs0612	*cusD*	-2.62	1	ECs2296	*ynfH*	-2.03	6
ECs0613	*ybdE*	-2.70	1	ECs2362	*lhr*	-2.16	1
ECs0670	*dacA*	-2.32	1	ECs2367	*purR*	-2.51	1
ECs0681	*ybeL*	2.78	2	ECs2431	*yniA*	3.52	2
ECs0773	*tolR*	-2.29	1	ECs2433	*yniC*	2.44	3
ECs0782	*aroG*	-3.09	1	ECs2445	*osmE*	2.33	2
ECs0800	*ybhC*	-2.13	1	ECs2510	*yeaV*	2.06	3
ECs0814	O157	-2.15	1	ECs2692	*yodD*	2.96	2
ECs0869	*ybhQ*	3.14	2	ECs2714	*espJ*	-2.01	1
ECs0890	*dps*	2.64	2	ECs2737	*pchC*	3.43	2
ECs0943	*artJ*	-2.81	1	ECs2814	*yeeD*	-2.09	1
ECs0966	*cspD*	2.75	2	ECs2816	*yeeF*	-2.55	1
ECs0968	*clpA*	2.89	3	ECs2839	O157	-2.23	1
ECs0986	*pflB*	-2.61	6	ECs2840	*wbdP*	-2.14	1
ECs1008	*ycbB*	2.15	2	ECs2847	O157	-2.25	1
ECs1029	*pyrD*	-2.88	1	ECs3038	*yeiT*	2.14	3
ECs1037	*rmf*	2.24	2	ECs3060	*fruK*	-2.13	1
ECs1072	O157	-2.13	5	ECs3061	*fruB*	-2.38	1
ECs1091	O157	3.24	2	ECs3136	*yfaX*	-2.04	1
ECs1137	*yccC*	-2.27	1	ECs3149	*menC*	-2.06	1
ECs1138	*yccY*	-2.78	1	ECs3180	*ackA*	-2.33	6
ECs1139	*yccZ*	-2.74	1	ECs3194	*argT*	4.08	2
ECs3196	*purF*	-2.94	1	ECs4188	*hopD*	2.38	2
ECs3197	*cvpA*	-3.35	1	ECs4192	*rpsG*	-2.57	1
ECs3212	*mepA*	-2.29	1	ECs4193	*rpsL*	-2.67	1
ECs3287	*ptsH*	-2.66	1	ECs4204	*yheT*	-2.00	1
ECs3296	*cysP*	-2.17	1	ECs4249	*yhgF*	-2.53	1
ECs3361	*purM*	-4.24	1	ECs4294	*yhhA*	2.48	2
ECs3387	*sseA*	2.18	2	ECs4343	*nikA*	-3.22	1
ECs3403	*hcaR*	2.50	3	ECs4347	*nikE*	-2.38	1
ECs3423	*purL*	-2.45	1	ECs4366	*yhiO*	4.26	2
ECs3448	*trxC*	2.24	2	ECs4481	*lldP*	2.39	2
ECs3463	*tyrA*	3.67	3	ECs4490	*gpmI*	-3.35	1
ECs3464	*aroF*	3.30	3	ECs4512	*rpmB*	-2.38	1
ECs3471	*yfjA*	-3.58	1	ECs4553	O157	-2.10	1
ECs3546	*emrR*	-3.47	1	ECs4575	*escC*	-2.00	1
ECs3559	*srlA_1*	2.08	3	ECs4632	*yidA*	-2.02	1
ECs3595	*rpoS*	2.80	2	ECs4638	*rpmH*	-2.47	1
ECs3606	*cysD*	-2.59	1	ECs4639	*rnpA*	-2.92	1
ECs3640	*pyrG*	-2.98	1	ECs4679	*atpE*	-2.33	1
ECs3659	*fucO*	2.17	3	ECs4716	*rho*	-2.56	5
ECs3675	*argA*	-4.09	1	ECs4720	*wecC*	-2.34	1
ECs3677	*recB*	-2.17	1	ECs4741	*xerC*	-2.08	1
ECs3701	*yqeF*	2.50	3	ECs4756	*yigL*	-2.18	1
ECs3743	*ygeW*	2.08	2	ECs4759	*metE*	-2.76	1
ECs3749	*yqeC*	2.45	2	ECs4791	*glnL*	-2.80	1
ECs3799	O157	-2.69	6	ECs4870	*metF*	-3.65	1
ECs3800	O157	-2.43	6	ECs4885	*ppc*	-3.62	1
ECs3802	O157	-2.43	6	ECs4887	*argC*	-3.99	1
ECs3811	*yggG*	2.28	2	ECs4888	*argB*	-3.62	1
ECs3818	*metK*	-3.03	1	ECs4893	O157	2.09	2
ECs3840	*nupG*	2.44	2	ECs4907	*rplA*	-3.02	1
ECs3931	*glgS*	2.94	2	ECs4908	*rplJ*	-2.64	1
ECs4052	*argG*	-2.68	1	ECs4909	*rplL*	-2.60	1
ECs4103	*rpsI*	-2.28	1	ECs4929	*purH*	-4.14	1
ECs4132	*yhdG*	-2.54	1	ECs4931	*metA*	-3.78	1
ECs4150	*smg*	2.24	2	ECs4932	*aceB*	5.35	2
ECs4156	*mscL*	2.01	2	ECs5004	O157	2.14	2
ECs4161	*rpsD*	-2.17	1	ECs5046	*yjcD*	-3.81	1
ECs4162	*rpsK*	-2.36	1	ECs5051	*acs*	5.55	2
ECs4164	*rpmJ*	-2.03	1	ECs5067	O157	2.21	2
ECs4165	*prlA*	-2.53	1	ECs5153	*purA*	-2.06	1
ECs5164	*yjfN*	4.54	2	ECs5240	*yjgR*	2.24	2
ECs5192	*cysQ*	2.01	2	ECs5259	O157	-2.09	1
ECs5231	*argI*	-3.29	1	ECs5354	*rob*	2.15	2
ECs5235	*valS*	-2.58	1	ECs5360	*yjjY*	2.03	2

**Table 3:** Significant ORFs (p value < 1 × 10^7^) with greater than 4-fold significant changes in expression from late exponential to early stationary phase (4.6 to 5.5 h)

Ecs no.	Gene^a^	Log2 change in expression	QT cluster	Ecs no.	Gene^a^	Log2 change in expression	QT cluster
ECs0009	*mogA*	2.27	2	ECs0526	*htpG*	-2.70	1
ECs0015	*dnaJ*	-2.32	1	ECs0527	*adk*	-3.22	1
ECs0026	*rpsT*	-4.14	1	ECs0540	O157	3.52	2
ECs0028	*ribF*	-2.36	1	ECs0541	O157	4.11	2
ECs0029	*ileS*	-2.81	1	ECs0552	*ybbK*	3.00	2
ECs0030	*lspA*	-2.38	1	ECs0561	*ybbD*	2.16	2
ECs0032	*lytB*	-2.20	1	ECs0615	*ybdG*	-2.36	1
ECs0036	*carB*	-3.28	1	ECs0619	*ybdK*	2.98	2
ECs0052	*ccdA*	2.41	2	ECs0645	*ahpF*	-2.11	1
ECs0078	*leuA*	2.55	un	ECs0646	*ybdQ*	3.35	3
ECs0108	*guaC*	-2.45	1	ECs0656	*citD*	2.79	2
ECs0118	*aceE*	-3.27	1	ECs0864	*ybhL*	2.61	2
ECs0119	*aceF*	-2.55	1	ECs0866	*ybhN*	2.27	2
ECs0123	*yacL*	3.17	2	ECs0881	*ybiI*	3.35	2
ECs0128	*gcd*	2.69	2	ECs0896	*ybiS*	-2.07	1
ECs0168	*dapD*	-3.16	1	ECs0915	*yliG*	-2.77	1
ECs0172	*tsf*	-4.58	1	ECs0919	*dacC*	2.40	2
ECs0173	*pyrH*	-3.15	1	ECs0934	*potF*	2.12	2
ECs0182	*fabZ*	-2.20	1	ECs0944	*artM*	-2.40	1
ECs0199	*yaeC*	-3.14	1	ECs0963	*ybjX*	-2.08	1
ECs0200	*yaeE*	-2.63	1	ECs0974	*lrp*	-3.19	1
ECs0201	*abc*	-3.32	1	ECs0979	*dmsA*	-3.37	4
ECs0216	O157	2.02	2	ECs0989	*ycaP*	2.56	2
ECs0248	*fadE*	4.57	2	ECs0990	*serC*	-3.29	1
ECs0253	*dinJ*	2.46	2	ECs0991	*aroA*	-4.24	1
ECs0269	*proB*	-2.11	1	ECs1007	*mukB*	-2.12	1
ECs0383	*yahO*	4.53	2	ECs1013	*asnS*	-2.74	1
ECs0384	*prpR*	3.63	2	ECs1014	*pncB*	-2.37	1
ECs0390	*codA*	-2.78	1	ECs1022	*ycbR*	-3.16	1
ECs0415	*afuA*	5.21	2	ECs1048	*-*	2.44	2
ECs0434	*psiF*	3.83	2	ECs1050	*yccV*	3.88	2
ECs0458	*yajC*	-2.33	1	ECs1158	*agp*	3.47	2
ECs0459	*secD*	-2.40	1	ECs1159	*yccJ*	2.12	2
ECs0490	*tig*	-2.96	1	ECs1211	O157	-3.29	1
ECs0497	*ybaW*	2.93	2	ECs1266	*phoH*	7.69	2
ECs0502	*mdlA*	-3.80	1	ECs1351	*terZ*	-2.58	1
ECs0503	*mdlB*	2.30	2	ECs1355	*terD*	-2.53	1
ECs0504	*glnK*	-3.73	1	ECs1356	*terE*	-2.90	1
ECs0507	*ybaY*	3.19	2	ECs1423	*-*	2.34	2
ECs0509	*ybaA*	2.56	2	ECs1438	*yceP*	4.78	2
ECs0518	*aefA*	2.12	2	ECs1466	*yceD*	-2.86	1
ECs1467	*rpmF*	-2.36	1	ECs2307	*ydgG*	-2.69	1
ECs1500	*potC*	-2.14	1	ECs2308	*pntB*	-2.80	1
ECs1603	*purB*	-3.58	1	ECs2314	*rstA*	-2.63	1
ECs1605	*ycfB*	-2.43	1	ECs2319	*manA*	-2.47	1
ECs1663	*ompT*	-2.72	1	ECs2346	*tyrS*	-2.97	1
ECs1683	*ycgB*	5.36	2	ECs2385	*ynhG*	3.80	2
ECs1692	*ymgE*	2.35	2	ECs2387	*sufS*	2.88	2
ECs1705	*-*	2.55	2	ECs2389	*sufC*	3.58	2
ECs1722	*chaB*	2.85	2	ECs2390	*sufB*	2.93	2
ECs1729	*narG*	-2.70	4	ECs2391	*sufA*	3.68	2
ECs1743	*oppA*	-2.75	1	ECs2397	*-*	2.44	2
ECs1747	*oppF*	-2.11	1	ECs2423	*rplT*	-3.11	1
ECs1831	*yciG*	2.91	2	ECs2430	*ydiZ*	2.09	2
ECs1848	*-*	2.73	2	ECs2450	*ydjS*	3.94	2
ECs1849	*acnA*	4.07	2	ECs2451	*astB*	4.11	2
ECs1858	*yciR*	3.14	2	ECs2452	*astD*	5.88	2
ECs1874	*ycjK*	4.73	2	ECs2453	*astA*	4.62	2
ECs1875	*ycjL*	5.14	2	ECs2454	*astC*	6.97	2
ECs1914	*ydaA*	2.88	2	ECs2460	*-*	-2.03	1
ECs1997	*ynaF*	3.42	2	ECs2463	*ynjE*	-4.04	1
ECs2028	*ydcJ*	3.64	2	ECs2467	*gdhA*	-5.15	1
ECs2036	*ydcL*	3.78	2	ECs2491	*yeaF*	-2.61	1
ECs2044	*ydcS*	5.22	2	ECs2492	*yeaG*	4.08	2
ECs2045	*ydcT*	5.23	2	ECs2511	*yeaW*	2.43	2
ECs2054	*ydcU*	2.59	2	ECs2514	*fadD*	2.50	2
ECs2082	*adhP*	3.62	2	ECs2519	*yoaC*	2.05	2
ECs2084	*rpsV*	2.45	2	ECs2527	*manX*	-2.95	1
ECs2086	*osmC*	2.01	2	ECs2528	*manY*	-3.21	1
ECs2091	*-*	3.38	2	ECs2546	*ydjX*	4.09	2
ECs2092	*-*	3.70	2	ECs2560	*eda*	-2.65	1
ECs2113	O157	-3.24	1	ECs2574	*yebC*	-2.87	1
ECs2118	*ydeV*	3.60	2	ECs2575	*ntpA*	-2.15	1
ECs2119	*ydeW*	2.54	2	ECs2586	*argS*	-2.77	1
ECs2120	*-*	4.17	2	ECs2609	*araF*	3.61	2
ECs2126	*tam*	2.83	2	ECs2613	*ftn*	-3.88	1
ECs2145	*ydeI*	3.74	2	ECs2614	*yecH*	-2.94	6
ECs2146	*ydeJ*	2.05	2	ECs2668	*yedE*	-2.15	1
ECs2150	*ydfZ*	-5.10	4	ECs2669	*yedF*	-2.78	1
ECs2281	O157	2.30	2	ECs2670	*yedK*	2.55	2
ECs2292	*-*	3.22	2	ECs2693	*-*	2.62	2
ECs2295	*ynfG*	-2.81	6	ECs2785	*erfK*	2.37	2
ECs2820	*hisG*	-2.80	1	ECs3458	*yfiO*	-2.61	1
ECs2821	*hisD*	-2.57	1	ECs3465	*yfiL*	3.81	2
ECs2822	*hisC*	-2.52	1	ECs3469	*rplS*	-4.28	1
ECs2824	*hisH*	-2.22	1	ECs3470	*trmD*	-5.46	1
ECs2837	*wbdQ*	-2.35	1	ECs3472	*rpsP*	-5.57	1
ECs2888	*yegP*	4.47	2	ECs3520	*ygaT*	4.88	2
ECs2892	*yegS*	3.38	2	ECs3522	*gabD*	2.53	2
ECs2900	*fbaB*	2.45	2	ECs3523	*gabT*	3.14	2
ECs2920	*metG*	-2.59	1	ECs3533	*ygaM*	3.32	2
ECs3022	*yohC*	6.12	2	ECs3554	*alaS*	-2.20	1
ECs3029	O157	4.04	2	ECs3605	*cysN*	-3.19	1
ECs3030	O157	3.38	2	ECs3617	*cysH*	-2.76	1
ECs3053	*yeiJ*	-2.67	1	ECs3619	*cysJ*	-4.11	1
ECs3055	*yeiL*	2.38	2	ECs3655	*ygdH*	2.14	2
ECs3073	*yejG*	2.91	2	ECs3669	*-*	2.28	2
ECs3077	*rplY*	-3.64	1	ECs3742	*ygeV*	3.64	2
ECs3078	*yejK*	-2.55	1	ECs3750	*ygfJ*	4.14	2
ECs3102	*ada*	2.24	2	ECs3761	*-*	2.76	2
ECs3114	*gyrA*	-3.38	1	ECs3762	*lysS*	-2.92	1
ECs3181	*pta*	-3.50	1	ECs3771	*-*	3.52	2
ECs3224	*fadJ*	4.26	2	ECs3772	*yqfB*	-2.02	1
ECs3225	*fadI*	4.50	2	ECs3784	*serA*	-3.66	1
ECs3227	*fadL*	5.39	2	ECs3810	*tktA*	-4.16	1
ECs3230	*yfdC*	2.55	2	ECs3815	*yqgB*	-2.40	1
ECs3259	*yfdZ*	3.57	2	ECs3833	*ansB*	-2.31	4
ECs3271	*-*	3.06	2	ECs3896	*yqhE*	2.86	2
ECs3272	*nupC*	-2.08	1	ECs3904	*ygiS*	-2.33	1
ECs3278	*gltX*	-2.47	1	ECs3948	*rpsU*	-3.14	1
ECs3286	*cysK*	-3.68	1	ECs3955	*ygjG*	5.55	2
ECs3293	*cysA*	-2.71	1	ECs3963	*ygjL*	4.06	2
ECs3302	*yfeX*	-2.24	1	ECs3979	*yqjC*	2.12	2
ECs3322	*-*	2.10	2	ECs3980	*yqjD*	2.28	2
ECs3323	*-*	2.15	2	ECs4033	*yraR*	2.71	2
ECs3338	*purC*	-5.04	1	ECs4034	*yhbO*	3.67	2
ECs3339	*nlpB*	-2.13	1	ECs4040	*yhbV*	-3.40	1
ECs3369	*guaA*	-2.99	1	ECs4064	*rpmA*	-3.29	1
ECs3397	*-*	2.55	2	ECs4068	*murA*	-3.01	1
ECs3401	*csiE*	3.77	2	ECs4069	*yrbA*	-2.73	1
ECs3409	*yphA*	3.07	2	ECs4091	*gltB*	-3.19	1
ECs3434	*lepB*	-2.08	1	ECs4092	*gltD*	-3.80	1
ECs3445	*yfiD*	-6.36	6	ECs4104	*rplM*	-4.05	1
ECs4112	*yhcO*	3.56	2	ECs4404	*yhjG*	2.50	2
ECs4127	*accB*	-2.57	1	ECs4427	O157	2.20	2
ECs4128	*accC*	-3.51	1	ECs4433	*yhjY*	3.88	2
ECs4133	*fis*	-3.76	1	ECs4438	*yiaE*	-2.49	1
ECs4159	*rplQ*	-3.99	1	ECs4440	*yiaG*	2.73	2
ECs4160	*rpoA*	-4.11	1	ECs4449	*xylF*	2.34	2
ECs4163	*rpsM*	-3.05	1	ECs4463	*yiaW*	2.75	2
ECs4166	*rplO*	-3.63	1	ECs4464	*aldB*	3.02	2
ECs4167	*rpmD*	-3.70	1	ECs4473	*yibH*	3.78	2
ECs4168	*rpsE*	-3.75	1	ECs4475	*mtlA*	2.84	2
ECs4169	*rplR*	-3.75	1	ECs4511	*rpmG*	-3.21	1
ECs4170	*rplF*	-3.62	1	ECs4514	*dfp*	-2.28	1
ECs4171	*rpsH*	-4.00	1	ECs4554	*espB*	-2.41	1
ECs4172	*rpsN*	-3.13	1	ECs4555	*espD*	-2.60	1
ECs4173	*rplE*	-4.26	1	ECs4611	*ilvN*	4.84	2
ECs4174	*rplX*	-4.14	1	ECs4612	*ilvB*	4.41	2
ECs4175	*rplN*	-4.46	1	ECs4633	*yidB*	3.85	2
ECs4176	*rpsQ*	-4.30	1	ECs4672	*glmU*	-2.20	1
ECs4177	*rpmC*	-4.12	1	ECs4674	*atpD*	-2.97	1
ECs4178	*rplP*	-3.60	1	ECs4675	*atpG*	-3.77	1
ECs4179	*rpsC*	-5.07	1	ECs4676	*atpA*	-4.02	1
ECs4180	*rplV*	-4.74	1	ECs4677	*atpH*	-4.50	1
ECs4181	*rpsS*	-4.14	1	ECs4678	*atpF*	-4.08	1
ECs4182	*rplB*	-4.83	1	ECs4680	*atpB*	-3.27	1
ECs4183	*rplW*	-5.75	1	ECs4684	*mioC*	-2.10	1
ECs4184	*rplD*	-6.50	1	ECs4686	*asnA*	-3.68	1
ECs4185	*rplC*	-4.69	1	ECs4708	*ilvC*	-3.31	1
ECs4186	*rpsJ*	-5.93	1	ECs4757	*yigM*	-2.00	1
ECs4189	*-*	3.24	2	ECs4758	*metR*	-3.26	1
ECs4191	*fusA*	-3.27	1	ECs4760	*-*	3.29	3
ECs4210	*argD*	-2.36	1	ECs4774	*fadB*	6.58	2
ECs4212	*fic*	4.44	2	ECs4783	*dsbA*	-2.15	1
ECs4213	*yhfG*	4.13	2	ECs4785	*yihG*	2.17	2
ECs4232	*aroK*	-3.21	1	ECs4792	*glnA*	-5.34	1
ECs4244	*yhgE*	-3.33	1	ECs4793	*yihK*	-2.43	1
ECs4278	*asd*	-2.55	1	ECs4847	*yiiS*	2.66	2
ECs4299	*ugpB*	6.46	2	ECs4869	*metL*	-2.30	1
ECs4348	*yhhG*	-2.67	1	ECs4884	*yijP*	-3.62	1
ECs4398	*yhjA*	-2.20	6	ECs4889	*argH*	-4.04	1
ECs4399	*treF*	3.03	2	ECs4898	*murI*	-2.51	1
ECs4402	*yhjD*	2.51	2	ECs4906	*rplK*	-4.80	1
ECs4910	*rpoB*	-2.90	1	ECs5123	*groES*	-3.66	1
ECs4911	*rpoC*	-2.86	1	ECs5130	*blc*	3.64	2
ECs4914	*thiG*	-2.22	1	ECs5176	*rpsF*	-4.84	1
ECs4915	*thiF*	-2.80	1	ECs5177	*priB*	-5.00	1
ECs4916	*thiE*	-2.34	1	ECs5178	*rpsR*	-4.46	1
ECs4917	*thiC*	-2.35	1	ECs5179	*rplI*	-3.90	1
ECs4928	*purD*	-4.58	1	ECs5185	*fklB*	-2.32	1
ECs4933	*aceA*	4.98	2	ECs5194	*ytfJ*	4.59	2
ECs4934	*aceK*	2.90	2	ECs5205	*ytfQ*	6.29	2
ECs4990	O157	2.13	2	ECs5206	*-*	2.57	3
ECs4991	O157	2.12	2	ECs5207	*ytfT*	3.32	2
ECs5007	*lysC*	-3.43	1	ECs5208	*yjfF*	2.59	2
ECs5038	*pfkA*	2.14	2	ECs5211	*yjgA*	2.20	2
ECs5039	*yjbR*	2.21	2	ECs5213	*cybC*	2.20	2
ECs5049	*actP*	4.17	2	ECs5222	*pyrB*	-2.95	1
ECs5050	*yjcH*	3.78	2	ECs5253	O157	2.70	2
ECs5089	*phnB*	4.22	2	ECs5332	*yjjG*	3.09	2
ECs5109	*yjdJ*	3.07	2				

^a^Genes with "O157" indicate that these are O157-specific ORFs.

Expression of *rpoS*, encoding the stationary phase sigma factor, increased 7 fold during the transition point and the RpoS activator, *dksA*, increased 4.3 fold at the same interval (Table 2). Increased expression of genes known to be regulated by RpoS, such as *bolA, dps, osmE, osmC, adhP *and *ugpB *occurred at the stationary phase transition point (Tables 2 and 3, Fig. 3; QTC 2). Gene set enrichment analysis also indicated that these genes (members of the adaptation to atypical conditions group) were significantly enriched in the stationary phase time points compared to exponential phase (Additional File 4). ORFs encoding toxin-antitoxin systems, such as *dinJ-yafQ *(Table 3, Fig. 3; QTC 2) and *chpB-chpS *(Fig. 3; QTC 3), were upregulated at the stationary phase transition point. The greatest increase in transcriptional level for an upregulated gene (45-fold) during the stationary phase transition (4.6 to 5 h interval) was *acs*, encoding acetyl CoA synthetase (Table 2). Acs expression is controlled in part by RpoS [46]. Also induced at a very high level during the transition point into stationary phase were *aceB *(40 fold) and *aceA *(32 fold), which encode components of the glyoxalate shunt. Increased expression of acetyl CoA synthetase as well as genes of the glyoxalate shunt is consistent with the hypothesis that cells are scavenging acetate produced during rapid growth on glucose in exponential phase.

There are a variety of O157-specific genes that were downregulated in the transition to stationary phase include those encoding the O157 LPS antigen (ECs2839, ECs2840, and ECs2847) with a greater than 4 fold decrease in expression (Table 3, Fig. 3; QTC 1) as well as ECs2835, ECs2836, ECs2838, ECs2841, ECs2844, and ECs2845 (1.6 to 3 fold decrease) from that genomic region. ECs2113, part of the F9 fimbrial operon [47], decreased in expression from exponential to stationary phase (Table 3). Other ORFs in this operon, ECs2107-2112, also decreased significantly (~1.5 fold) upon entry into stationary phase. O157-specific ORFs that increased in stationary phase included ECs2182, ECs2737, and ECs5067, which encode putative transcriptional regulators. ECs2737 (*pchC) *encodes a *perC*-like homolog reported to regulate LEE transcription [48]. The recently identified chromosomal CcdAB toxin-antitoxin system [49], encoded by ECs0052 and ECs0053, increased 5.3-fold from the transition point to early stationary phase (Table 3, Fig. 3; QTC 2). ECs0415, *afuA*, encoding a periplasmic ferric iron binding protein, had the greatest increase in expression of the O157-specific ORFs, with a 37-fold increase during the transition point (Table 3, Fig. 3; QTC 2).

### Genes with transient expression at the stationary phase transition point

A subset of 120 ORFs were expressed transiently from late exponential to early stationary phase. These ORFs changed significantly during the 4.6 to 5 h time interval, and then again during the 5 to 5.5 h interval, but in the opposite direction (Fig. 3; QTC 3, 11, and 12). Genes with this pattern of transcription included many involved in nutrient scavenging and turnover. Genes encoding both the anaerobic (*glpABC*) and aerobic (*glpD*) *sn*-glycerol 3 phosphate dehydrogenase increased >4 fold during the transition into stationary phase, and then decreased significantly in early stationary phase (Table 4). This pattern is consistent with the general model that the *glp *metabolic system serves as a salvage pathway for glycerol derived from degradation of phospholipids and triacylglycerol. The *murPQ *operon was expressed transiently, with a 5.3 fold increase followed by a 2 fold decrease in early stationary phase. The products of *murPQ *are involved in peptidoglycan turnover [50] suggesting that membrane components are being recycled and utilized for energy. A dicarboxylate transporter, *dctA*, was induced 24-fold upon entry into stationary phase, then decreased 4-fold in early stationary phase (Table 4). Genes encoding transport and binding proteins for carbohydrates, sugar alcohols and acids were significantly enriched at the stationary phase transition point compared to mid-exponential phase, also indicating the release of glucose-dependent catabolite repression (Additional File 4). Genes encoding salvage and scavenging pathways are also induced during glucose-limited growth [51,52] (Additional File 1).

**Table 4:** Significant ORFs (p value < 1 × 10^7^) with transient expression at the stationary phase transition point with expression changes greater than 4-fold.

Ecs no.	gene	log2 change in expression 4.6–5 h	log2 change in expression 5–5.5 h	QT cluster
ECs0057	*pdxA*	-2.43	0.66	1
ECs0287	O157	-1.09	2.91	2
ECs0358	*betB*	3.07	-1.25	3
ECs0359	*betI*	2.99	-1.48	3
ECs0418	O157	2.19	-0.64	3
ECs0486	*cyoA*	2.77	-1.08	10
ECs0746	*sdhC*	2.93	-1.74	10
ECs0747	*sdhD*	2.55	-1.59	10
ECs0748	*sdhA*	2.76	-1.38	10
ECs0778	*nadA*	-2.57	0.76	1
ECs0946	*artI*	-2.66	0.40	5
ECs0987	*focA*	-3.38	1.11	6
ECs0994	*rpsA*	-3.78	0.66	1
ECs1261	*putP*	2.80	-0.92	10
ECs1741	*adhE*	-4.01	1.17	6
ECs1744	*oppB*	-2.05	1.36	7
ECs1772	O157	-0.68	2.22	2
ECs2383	*pykF*	-2.42	1.49	un
ECs2387	*sufS*	0.71	2.17	2
ECs2481	*ydjH*	-2.44	0.84	6
ECs2482	*ydjI*	-2.18	0.81	6
ECs2486	*yeaC*	3.00	-0.57	2
ECs2487	*yeaA*	3.08	-0.60	3
ECs3042	*mglB*	3.96	-1.13	3
ECs3043	*galS*	2.14	-0.87	3
ECs3117	*nrdA*	1.83	-3.64	11
ECs3125	*glpT*	1.65	-2.22	4
ECs3126	*glpA*	3.61	-2.78	3
ECs3127	*glpB*	2.59	-2.15	3
ECs3128	*glpC*	2.92	-2.94	un
ECs3142	*yfbF*	-2.54	1.59	12
ECs3143	*yfbG*	-2.50	1.03	5
ECs3144	*yfbH*	-2.66	0.98	5
ECs3299	*yfeU*	2.39	-0.89	3
ECs3300	*yfeV*	2.28	-0.99	3
ECs3305	*ypeA*	2.05	-0.81	3
ECs3393	*hscB*	-1.37	2.51	7
ECs3748	*yqeB*	2.23	-0.78	3
ECs4109	*mdh*	2.05	-1.40	un
ECs4269	*glpD*	4.17	-1.82	3
ECs4408	*dctA*	4.59	-1.93	3
ECs4498	*rfaF*	-2.10	0.85	12
ECs4705	*ilvD*	-2.03	1.03	12
ECs4726	*yifM*	3.51	-3.62	un
ECs4750	*yigI*	2.77	-1.14	10
ECs4840	*fieF*	3.76	-3.29	3
ECs4851	*glpK*	3.54	-3.73	4
ECs4852	*glpF*	5.22	-4.10	3
ECs4918	*yjaE*	4.21	-1.58	3
ECs5298	*yjiM*	0.71	-2.14	8
ECs5313	*yjiY*	1.38	-3.28	un

Only 12 O157-specifc ORFs were expressed transiently upon entry into stationary phase. ECs1772, a putative intestinal colonization factor, decreased upon entry into stationary phase, then significantly increased in expression (>4 fold increase from 5 to 5.5 h) (Table 4).

### Genes with a significant change in expression in early stationary phase

The interval with the second largest number of significantly modulated ORFs was in early stationary phase, during the 5 to 5.5 h interval. A number of these ORFs that had significant increases in expression levels during this interval are transcriptional regulators, such as the AraC-like regulators *gadW *and *gadX*, involved in regulation of the glutamate decarboxylase system of acid resistance (Table 5). Genes encoding DNA binding proteins, such as *hha *and *cbpA*, also increased in transcription in early stationary phase. Components of the glycerol metabolism pathway were activated in early stationary phase, including *ugpC *and *ugpE*, components of the glycerol 3 phosphate transporter, and *ugpQ*, the cytosolic glycerophosphoryl diester phosphodiesterase (Table 5).

**Table 5:** Significant ORFs (p value < 1 × 10^7^) with greater than 4-fold expression changes in early stationary phase.

Ecs no.	gene	log2 change in expression 5–5.5 h	QT cluster	Ecs no.	gene	log2 change in expression 5–6 h	QT cluster
ECs0342	*ykgC*	2.30	2	ECs0568	*gcl*	4.04	2
ECs0513	*hha*	2.55	2	ECs0569	*gip*	3.56	2
ECs0514	*ybaJ*	2.16	2	ECs0570	*ybbQ*	4.89	2
ECs0732	*ybgA*	2.27	2	ECs0571	*ybbV*	3.27	2
ECs0733	*phrB*	2.15	2	ECs2095	*yddV*	4.27	2
ECs0769	*cydB*	-2.03	4	ECs2122	*ydeZ*	4.88	2
ECs0957	*poxB*	2.47	2	ECs2123	*yneA*	5.14	2
ECs0973	*trxB*	-2.25	1	ECs2124	*yneB*	4.46	2
ECs1155	*cbpA*	2.26	2	ECs2705	*yedU*	4.54	2
ECs1236	*lomW*	2.21	2				
ECs1756	*yciD*	-3.21	4				
ECs2047	*ydcV*	3.59	2				
ECs2048	*ydcW*	2.35	2				
ECs2386	*ynhA*	2.75	2				
ECs2388	*ynhC*	2.89	2				
ECs2493	*yeaH*	4.26	2				
ECs2529	*manZ*	-2.65	1				
ECs3395	*iscU*	2.61	2				
ECs3396	*yfhO*	2.04	2				
ECs4046	*rpsO*	-2.20	1				
ECs4295	*ugpQ*	2.20	2				
ECs4296	*ugpC*	2.60	2				
ECs4297	*ugpE*	2.12	2				
ECs4305	*livK*	-2.23	1				
ECs4395	*gadW*	2.10	2				
ECs4396	*gadX*	2.76	2				
ECs4534	*intL*	2.50	2				
ECs4673	*atpC*	-2.24	1				
ECs5108	*yjdI*	2.20	2				
ECs5269	*yjhT*	3.09	2				

### Expression of LEE island genes upon entry to stationary phase

**Figure 5:**
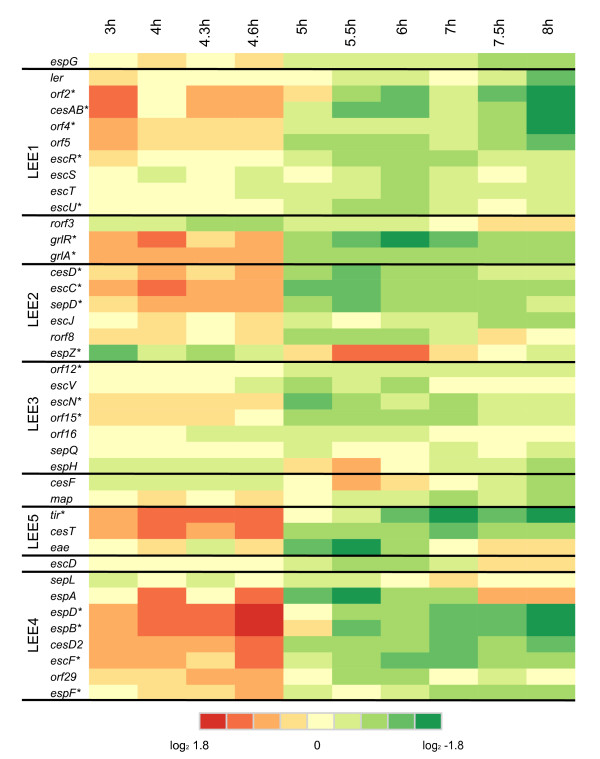
**Heatmap of gene expression over time for the ORFs of the LEE Pathogenecity Island**. Expression values determined from the ANOVA analysis are represented colorimetrically, with dark red representing expression = 1.8 and dark green representing expression = -1.8 on a log2 scale. ORFs marked with an asterisk denotes that a statistically significant change in expression levels of that ORF was detected for at least one time point.

Of the 41 LEE island ORFs, 23 had a significant (*p *< 0.00001) differences in expression in growth into stationary phase under minimal media (Fig. 5). Most of these ORFs were downregulated in transition to stationary phase (between 4.6 and 5 h). Three of these ORFs, *espB, espD*, and *espZ*, also had a significant change in expression in early stationary phase (5 h to 5.5 h). The greatest reduction in expression was observed in *espB *and *espD*, which both decreased 5.5- fold from late log to early stationary phase. Expression of *espA *also decreased > 5- fold over this interval, but the error variance for the *espA *probe was large and thus was not deemed significant. While the overall trend for the LEE island genes was a decline in expression during the transition to stationary phase, the exception was *espZ *which had a 3.2 fold increase in expression from late log to early stationary phase, indicating that this ORF may be activated by stationary phase associated regulators. The LEE ORFs as a group were found to be significantly enriched in the 3 h log-phase sample compared to the 5 h, 5.5 h, and 6 h stationary phase samples (Additional File 4).

**Figure 6:**
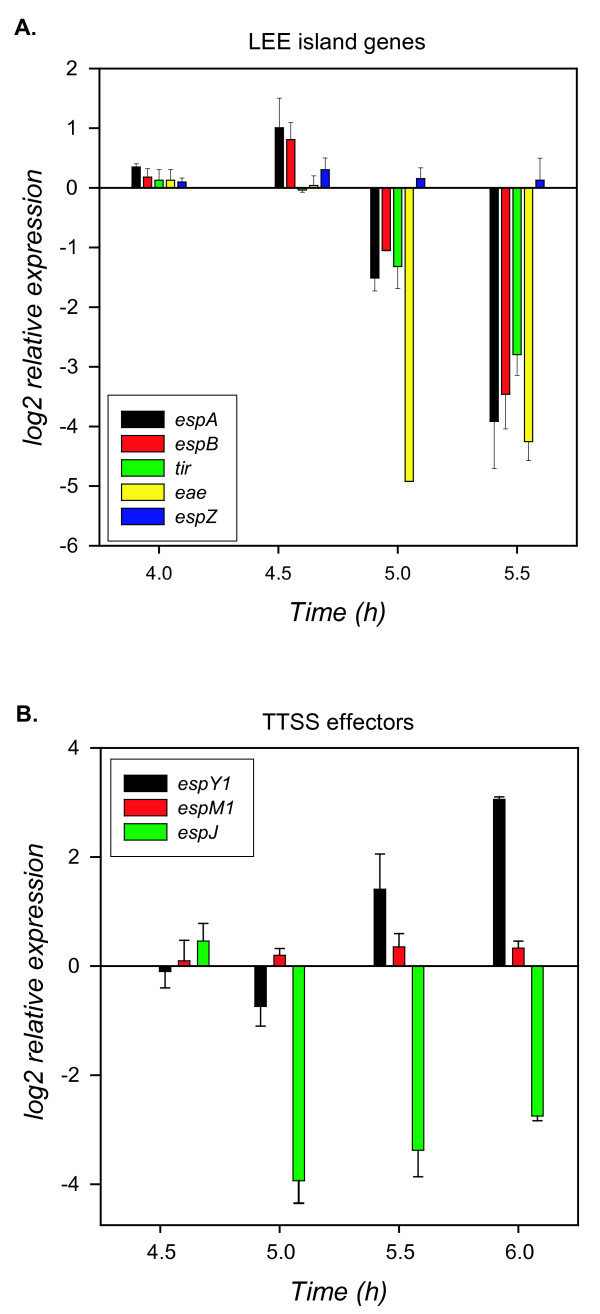
**Relative changes in expression for virulence-associated genes from the LEE Pathogenecity Island**. **A**. Expression differences of 5 genes transported through or adherence components of the LEE island as determined by Q-PCR. **B**. Expression differences of 3 genes encoding effectors (Esp) molecules transported through the LEE Type III Secretion System (TTSS) as determined by Q-PCR results. Average changes in expression for 2 independent replicate cultures were determined relative to the transcript level of each ORF at the 3 h mid-exponential phase time point.

Based on the array data, a significant change in expression could not be detected for *espA *or *eae *as a result of the high variability in signal for probes targeting those ORFs. Because *espB, espD *and *tir *significantly decreased in expression, we used Q-PCR to determine if expression levels changed for *espA *and *eae*. Q-PCR confirmed the array data for *espB*, and detected a 32-fold decrease in *espA *and a 16-fold decrease in *eae *transcript levels from 4.6 to 5.5 h (Fig. 6A). Overall, the Q-PCR data detected greater amounts of decrease in *espA, espB *(22- to 35-fold), *tir*, and *eae *(7- to 16-fold) expression compared to the array data (3.2-fold decrease for *tir *and *eae*, 5- to 7-fold decrease for *espA *and *espB*) for the 4.6 to 5.5 h interval. Transcript levels for *espZ *were estimated to increase 3.2-fold for the 4.6 to 5.5 h interval from the array data. Q-PCR for *espZ *found only a slight change (~1.2-fold) in expression over the time intervals tested (Fig. 6A). Whereas the Q-PCR measurements did not confirm the increase in expression determined from the array data for *espZ*, it is clear that *espZ *expression does not follow the same pattern as other genes on the LEE island.

### Expression changes of LEE effectors

More than 60 putative effectors that are translocated through the LEE-encoded TTSS were recently identified [53] and of these TTSS effectors, 33 were found here to have significant (*p *< 0.00001) changes in expression over time. A total of 7 TTSS effector genes (*nleA, nleG, espM1, espM2, nleG2-2, espZ*, and *nleG2-3*) amplified in expression between mid- and late- exponential phase (3 h to 4 h). At the stationary phase transition point, 11 ORFs increased in expression and 7 ORFs had a significant reduction in expression (*espJ, tccP, tir, espF, espB, espM2*, and *nleD*). In early stationary phase, eleven ORFs were upregulated, with the exception of *espB *(2.5 fold decrease). From late exponential to early stationary phase (4.6 h to 5.5 h), 8 ORFs had an increase in expression > 2- fold, with *espY1 *increasing 9 fold. Over this same time interval, 5 TTSS effector ORFs had a 2-fold or greater decrease in expression, with *espB *decreasing 5.3 fold (Table 6). Array data for the TTSS effectors *espY1 *and *espM1 *indicated an increase in expression from exponential to stationary phase, which was reflected in the Q-PCR data for these ORFs (Fig. 6B). Expression of the TTSS *espJ *decreased in stationary phase and a 21 -fold decrease in expression during the transition from log to stationary phase (4.6 to 5 h) was determined by Q-PCR (Fig. 6B).

**Table 6:** Significant changes in transcript levels of Type III secretion system (TTSS) effectors.

ECs number	Gene name	Log_2 _change in expression from 4.6 h to 5.5 h
ECs0061	*espY1*	3.03
ECs0073	*espY2*	1.24
ECs1825	*espM1*	1.47
ECs1994	*nleG2-2*	1.41
ECs2156	*nleG2-3*	1.63
ECs2229	*nleG2-4*	-1.15
ECs2714	*espJ*	-2.18
ECs2715	*tccP*	-1.39
ECs3488	*nleG6-3*	1.11
ECSs4554	*espB*	-2.41
ECs4561	*tir*	-1.72
ECs4571	*espZ*	1.69
ECs4643	*espL3*	1.77

### Significant increase of genes from the acid fitness region in stationary phase

**Figure 7:**
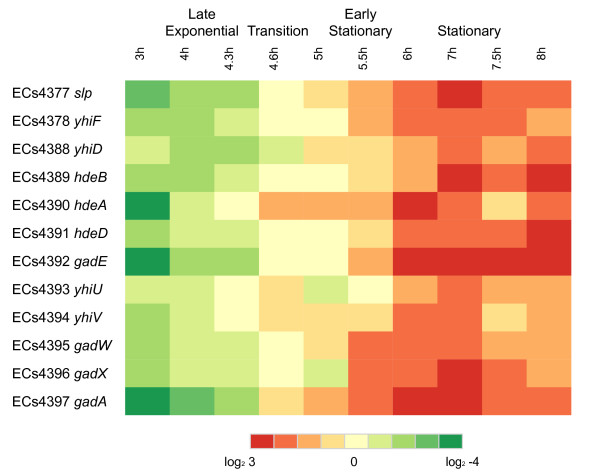
**Expression heat map of 12 genes from the acid fitness region (AFI)**. Expression values determined from the ANOVA analysis are represented colorimetrically, with dark red representing expression = 3 and dark green representing expression = -4 on a log2 scale. Over half of the AFI genes (7/12) had a significant increase in expression from mid- to late- exponential phase

A cluster of 12 genes located in the *E. coli *K-12 genome at position 3652706 to 3665603 bp has recently been termed the acid fitness island (AFI) [54]. In *E. coli *O157:H7 Sakai genome, the homologous region contains a 9 kb insertion between *yhiF *(ECs4378) and *yhiD *(ECs4388). This AFI genomic region contains multiple transcriptional regulators that control expression of the glutamate dependent acid resistance (GDAR) system, such as GadE, GadX, and GadW, that are also known to influence LEE expression [55,56]. The microarray comparisons demonstrate that the expression of the AFI genes increased significantly from exponential to stationary phase, and included some of the greatest increases in transcript level among the whole genome (Fig. 7). Transcript levels of *gadA *increased 111 fold and levels of *gadE *increased 85 fold from mid-exponential (3 h) to stationary phase (6 h). Over half of the AFI genes (7/12) had a significant increase in expression from mid- to late- exponential phase (Fig. 7). Transcript levels of *gadA, gadE*, and *slp *also increased significantly from 4.3 to 4.6 h, which is different from the expression profiles of the other known stationary phase activated genes that increase from 4.6–5 h. GSEA found the AFI ORFs as a group to be significantly enriched in all of the time points subsequent to the mid-exponential phase sample (Additional File 4).

### Increased expression of Shiga toxin genes upon entry into stationary phase

**Figure 8:**
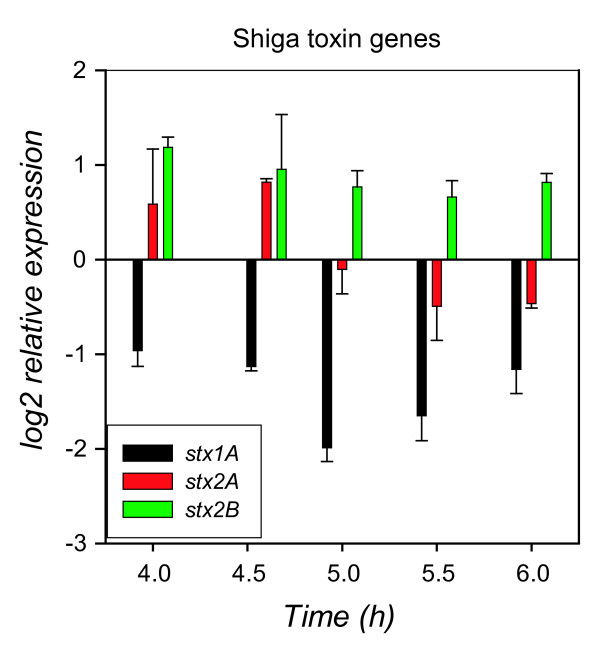
**Relative changes in expression of Shiga toxin genes**. Changes in expression of the phage-encoded toxin genes *stx1A, stx2A*, and *stx2B *as determined by Q-PCR. Average changes in expression for 2 independent replicate cultures were determined relative to the transcript level of each ORF at the 3 h mid-exponential phase time point.

More than 50% of Sp 15 ORFs and 60% of Sp 5 ORFs had significant (p < 0.00001) changes in expression over time into stationary growth phase. The genes encoding Shiga toxin 1, *stx1A *and *stx1B*, as well as those encoding Shiga toxin 2, *stx2A *and *stx2B*, had a significant difference in expression between mid- and late- log phase (3 to 4 h). Stx1 genes decreased ~1.8 fold over this interval, while Stx2 genes increased ~1.7 fold. Stx1 genes increased significantly in early stationary phase, with a ~4- fold increase from 5 to 6 h. A significant 2.5- fold increase for *stx2B *occurred at the transition point into stationary phase, but a concomitant increase was not observed for the *stx2A *ORF on the microarray. Q-PCR was used to monitor expression of *stx1A, stx2A*, and *stx2B *over time (Fig. 8). Expression of *stx1A *decreased 4-fold from mid-exponential phase to the stationary phase transition point, and then increased 2-fold during early stationary phase (5 to 6 h interval), which is similar to the array data. A 2-fold increase in expression from mid- to late- log phase was observed for *stx2B *using Q-PCR. While *stx2B *transcript levels remained higher in stationary phase compared to exponential phase, a subsequent increase during the stationary phase transition was not observed. Transcript levels of *stx2A *increased 1.5-fold from mid- to late- exponential phase, and decreased 1.5-fold from late exponential to stationary phase (Fig. 8). The ORFs encoding the phage replication factors O and P, as well as the CII regulatory protein and the Q antiterminator protein, increased in expression upon entry into stationary phase, but it is difficult to attribute these ORFs to a specific phage, as there are multiple copies of these ORFs throughout the genome.

### Expression changes in tellurite resistance genes and urease genes

**Figure 9:**
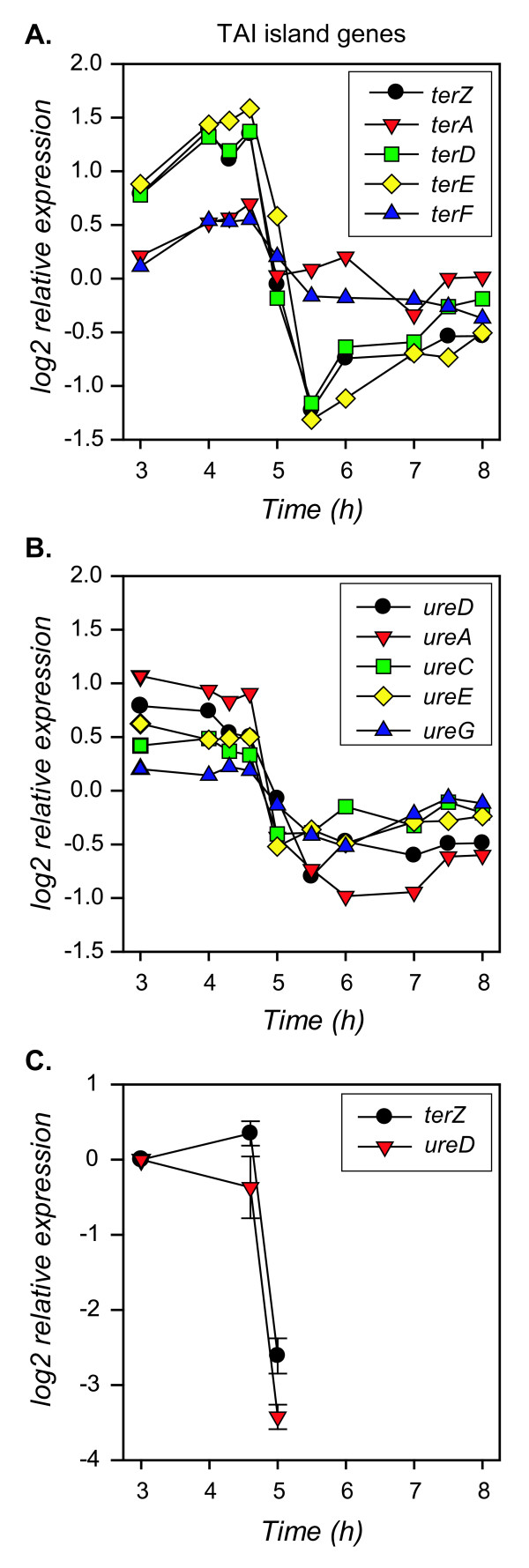
**Expression profiles of 12 ORFs encoded on the tellurite resistance island (TAI)**. Expression values determined from the ANOVA analysis are plotted over time for the **A**. tellurite resistance and **B**. the urease-encoding ORFs. **C**. Average relative expression of *terZ *and *ureD *was determined by Q-PCR of two independent replicate cultures.

Proteins involved in tellurite resistance are encoded on a pathogenicity island (TAI) along with genes encoding urease and are present in a single copy in *E. coli *O157:H7 Sakai [16]. Significant changes in expression over time were detected only for *terZ, terD*, and *terE *(Fig. 9A). Probes targeting *terB *and *terC *are not present on the array. Expression of these three genes increased significantly between mid- and late- exponential phase (~1.5 fold) and then decreased 5.9- to 7.4- fold during the transition into stationary phase and in early stationary phase (4.6 h to 5.5 h interval). These three ORFs were grouped into QT cluster 1. The *ureD, ureA, ureC, ureE*, and *ureG *ORFs had a significant reduction in expression from late exponential to early stationary phase (1.5 to 3 fold) and were grouped into QT cluster 1 (Fig. 9B). Also located on this genomic island is the adherence factor *iha *[57], but there is no significant change in *iha *mRNA over time. Quantitative real-time PCR targeting *terZ *and *ureD *confirmed the reduced expression of these ORFs from exponential to stationary phase, detecting an 8-fold decrease for *terZ *and *ureD *(Fig. 9C).

## Discussion

By monitoring global transcript levels over time, we have measured the numerous alterations in gene expression patterns of *E. coli *O157:H7 when growing exponentially and then entering stationary phase in minimal medium. *E. coli *O157:H7 responded to low O_2 _by increasing expression of genes encoding anaerobic electron acceptors and decreasing expression of genes encoding the TCA cycle. O157-specific ORFs, including *nleA *and *nleG*, which encode TTS effectors, were upregulated during this interval. As cells entered stationary phase, ~50% of the ORFs in the genome responded with significant changes in transcript level. The global trends include down regulation of some of virulence factors, such as the components of the TTSS and some of the TTSS effectors, in stationary phase. Other TTSS effectors and the genes encoding Shiga toxin increased in transcript level upon entry to stationary phase. Entry into stationary phase was characterized by the increase in expression of known stress and survival related genes, such as the osmotic stress proteins encoded by *osmB *and *osmC*, and the glutamate dependent acid resistance system encoded by *gadA *and *gadBC*.

As *E. coli *O157:H7 cells grew in the minimal medium, O_2 _levels in the medium decreased and were not replenished from the atmosphere (Fig. 4). The decrease in available oxygen was reflected in the transcriptome, with significant increases in genes encoding anaerobic electron acceptors, decrease in transcript levels of TCA cycle genes, and increase in expression of pyruvate formate lyase and concomitant decrease in expression of the pyruvate dehydrogenase complex. Decreased expression of *cyo *and *sdh *operons, with concurrent increased expression of the *cyd *operon was also observed during anaerobic growth of *E. coli *K-12 at pH 4.7 compared to aerobic growth at acidic pH [28] (Additional File 1). 5% (73/1239) of O157-specific ORFs also had significant changes in expression over the same time interval, which leads one to speculate that these O157-specific ORFs may be regulated by transcription factors that influence expression of respiratory genes, such as FNR and ArcA. Virulence factor expression in enteric pathogens, such as *Salmonella enterica *serovar Typhimurium and *Vibrio cholerae*, can be controlled by the redox state regulators FNR and ArcA [58-60]. A search for the FNR and ArcA binding sequences 500 bp upstream of all ORFs in the Sakai genome (using Pattern Search on coliBASE [61]) did not identify any of the ORFs that were significantly expressed or repressed in exponential phase. It may be that these O157-specific ORFs are regulated indirectly through FNR or ArcA, or are modulated in a completely different manner. Recently Ando et al. [62] demonstrated that anaerobic growth in the presence of the electron acceptors nitrate or TMAO accelerates maturation of the TTSS, independently of new protein synthesis. They found that the *narGHIJ *operon must be present for the maturation of the TTSS. Our findings indicate that the expression of this operon increases significantly in late exponential phase, which is consistent with the *narGHIJ *operon contribution to the maturation of the TTSS in this environment.

Much is known about regulation of LEE expression; multiple circuits of activation and repression have been identified. LEE expression can be induced in response to a number of environmental stimuli, and while common transcriptional regulators and signaling systems can activate LEE expression, they all typically act on Ler or the recently described GrlA and GrlR regulators located on the LEE island [63], which then alter expression of other LEE genes. As LEE expression is thought to be maximal under conditions similar to the intestinal environment [11], we would expect to see increased expression of LEE under these conditions compared to the minimal medium used here. We did observe similar growth-phase dependent changes in expression in the LEE genes that has also been observed for O157:H7 grown in rich medium. LEE expression is known to be maximal in late exponential phase [12,64]. Nakanishi et al. determined that LEE can be stimulated during nutrient downshifts through activation of ppGpp and DksA [10]. The pattern of *espB *expression observed during induction of LEE with ppGpp is similar to our observation, in that *espB *and *espA *expression increases from mid- to late-exponential phase, and then decreases in stationary phase (Fig. 5 and Fig. 6A). LEE transcription can also be stimulated by quorum sensing, via the auto-inducer 3 signaling system that activates *ler *[12]. The quorum sensing activated regulator QseA which activates LEE transcription [65] had a decrease in transcript levels from exponential to stationary phase (Fig. 3; QTC 1). Many of the common *E. coli *regulators that have been shown to repress LEE expression, such as Hha [66], SdiA [67], GadE [56], YhiF [56], and IHF [68,69], all increased in mRNA levels from exponential to stationary phase (Table 2, Fig. 3; QTC 2). Other LEE activators, such as PchC [48], had a significant increase in mRNA in stationary phase.

The decrease in expression of LEE genes from exponential to stationary phase that we observed is similar to the expression changes of LEE genes when O157:H7 is attached to red blood cells [34]. In comparing our dataset to the genes that were found to be significantly up- or down-regulated in O157:H7 attached to red blood cells, we found that of the 299 ORFs reported to be down regulated in attached cells, 185 had significant changes in expression over time, and 73% of these ORFs were classified in QT cluster 1 (Fig. 3), which includes the LEE genes. Of the 105 ORFs reported to be upregulated in attached cells, 89 had significant changes in expression over time, and 56% of these significant ORFs were placed in QT cluster 2 (Fig. 3), suggesting that many of the ORFs expressed or repressed during attachment to plasma membranes are also regulated by growth phase.

Not as much known about regulation of non-LEE encoded TTSS effector genes. The presence of some of the TTSS effectors in the genome is variable among O157 strains [70]. The TTSS effectors *tccP *and *espJ *are located on Sakai phage 14 (EDL-933 OI#79); *tccP *(*espFu*) is necessary for actin recruitment and A/E lesion formation in *E. coli *O157:H7, while *espJ *influences colonization dynamics [71-73]. Reading and colleagues recently determined that the AI-3, epinephrine, and norepinephrine response regulator QseF (*yfhA*) is an indirect transcriptional activator of *tccP *[74]. Our data indicate that as Sakai O157:H7 entered stationary phase, *qseF *mRNA levels decrease significantly (2 fold) (QTC 1) and *tccP-espJ *levels decreased significantly as well. This decrease in *tccP *and *espJ *transcript levels in stationary phase is in contrast to a previous study, where the activity of the *tccP-espJ *promoter was found to be the same in exponential vs. stationary phase cultures of EDL-933 in Dulbecco's Modified Eagle Medium as monitored by a *gfp *fusion [75]. Some of the TTSS effectors appear to respond to low O_2 _conditions, as 7 effectors increased significantly from mid- to late-exponential phase when oxygen levels decreased. Of the TTSS effectors with significant changes in expression over time, ~50% have decreased expression from exponential to stationary phase, while the other half have increased expression in stationary phase. Several of these TTSS effectors are predicted to be pseudogenes; one example is *espL3*, an ORF that was found to have a significant increase in expression at the stationary phase transition point. Many of the TTSS effectors with increased expression levels in stationary phase are members of the NleG family (Table 6), one of the largest families of TTS effectors that contain many duplications [53]. The utility of the increased expression of some of the TTS effectors in stationary phase is unclear and warrants further investigation.

The ability to resist low pH is a crucial adaptation and critical component of the low infectious dose of *E. coli *O157:H7. The GDAR system provides the greatest protection against low pH, allowing survival at levels of gastric acidify (e.g. pH 2.0) as long as glutamate is present in the environment [76]. Genes encoding the glutamate decarboxylase isozymes and the glutamate-gamma-aminobutyric acid antiporter are under tight control and can be activated via a number of environmental cues and transcriptional regulators [77], and many components of the system are located in the AFI region [54]. Also located on the AFI are *hdeA, slp*, and *yhiF*, which encode proteins involved in protecting cells from their metabolic by-products when the medium is acidified, and *yhiD *and *hdeD*, which are required for density-dependent acid resistance [78]. ORFs of the AFI are induced by a variety of stressful environments, including growth in the presence of acetate [79], anaerobic, low pH growth [28], during attachment to red blood cells [34], and in stationary phase [23]. Induction of the AFI ORFs as a response to multiple types of stresses highlights the importance of acid stress survival to the route of transmission for *E. coli*. Transcription factors located on the AFI, such as GadE and GadX, regulate a large number (>40) of ORFs outside of the GAD system in *E. coli *K-12 [54,80], and it is known that these factors serve as repressors of LEE transcription [55,56], but it is unknown what the effect of these regulators are on a genome-wide scale in *E. coli *O157:H7.

Chromosomal Toxin-antitoxin (TA) loci encode TA systems which are thought to induce reversible stasis, allowing survival during episodes of extreme nutritional stress [81,82]. Increased expression of TA loci in *E. coli *K-12 during growth transitions led to the development of a model integrating inhibition of all macromolecular synthesis within the stringent response model [23], while recent findings suggest that the chromosomal TA systems do not significantly influence bacterial fitness during nutrient limitation [83]. *E. coli *O157:H7 has some of the same TA loci as *E. coli *K-12; *dinJ-yafQ *[84], *chpAR, chpBS *[85], as well as the recently described chromosomal *ccdAB *system that is present in *E. coli *O157:H7 and not in *E. coli *K-12 [49]. CcdB encodes a DNA gyrase toxin [49], *chpB *encodes a translation inhibitor [86], and *yafQ *encodes an mRNA endoribonuclease [84]. Here we observed increased expression of the *dinJ-yafQ, chpBS*, and *ccdAB *TA loci upon entry to stationary phase, indicating that these systems are actively transcribed in stationary phase, but their utility remains to be elucidated.

Important questions remain about the factors regulating the Shiga toxins which are encoded by lambda-like bacteriophages [7]. These phages can be transferred between many *E. coli *strains in nature and were the principal virulence factors acquired as a key step in the evolution of *E. coli *O157:H7 [87]. How they become influenced in expression and regulated when acquired into a new genome is not fully understood. Early work described two types of Shiga toxin [88] encoded by Stx1 and Stx2, both located within the late operons of the Stx-encoding phages [89,90]. Stx1 expression is regulated in part by the iron-dependent transcriptional repressor, Fur [15]. Exposure of *E. coli *O157:H7 to low iron conditions result in an increase of *stx1 *transcription [14]. Our growth experiments indicate that the *stx1 *genes decrease in expression from mid- to late-exponential phase, at the same interval when O_2 _levels decrease (Fig. 8). Iron becomes more soluble as O_2 _concentration decreases, which could lead to the decreased *stx1 *expression. Stx1 expression can be induced at 2 other promoters in addition to the Fur binding site, those that require the phage anti-terminator Q [91] and the phage anti-terminator N [14]. Stx2 transcription is initiated at the late phage promoter and is dependent on the anti-repressor Q protein [13]. The production of Stx2 is part of the lytic cycle of the bacteriophage, and can be induced by antimicrobials [92,93]. Herold et al. found that phage genes were induced upon exposure of *E. coli *O157:H7 EDL-933 to norfloxacin; phage late genes and *stx2 *were induced 150-fold [35]. Levels of *stx2 *mRNA are higher in starved O157:H7 compared to exponentially growing cells [94], indicating an overall trend of *stx2 *expression induction by stress. Here we observed that *stx1 *expression increased in stationary phase and *stx2 *expression was higher in stationary phase compared to exponential phase (Fig. 8). Herold et al. also reported that *stx2A *is transcribed more efficiently than *stx2B *in O157:H7 EDL-933, and may be regulated at the posttranscriptional level [35]. This difference in transcription efficiency between *stx2A *and *stx2B *was not reflected in our data, as *stx2A *levels did not appear to increase as the *stx2B *levels did in stationary phase.

The genomic island termed the Tellurite and Adherence Island (TAI) contains genes encoding tellurite resistance, urease, and an adherence factor, *iha *[57]. The urease operon and the tellurite genes are thought to be ubiquitously distributed in EHEC strains [95,96] but is not present in O157:NM or O55:H7 strains [97]. Urease activity has been detected in relatively few EHEC strains. In O157:H7 Sakai, the urease genes have been detected at the transcript level, but a functional protein is not produced due to a stop codon present in *ureD *[98]. Saridakis et al. surveyed 25 O157:H7 strains that were isolated from humans, cattle, and pigs for *ureC *and the ability to resist low pH using the urease enzyme. *UreC *was detected in all of the isolates but there was no evidence for urease-mediated acid resistance [99]. Here we see that the genes encoding urease are expressed during exponential growth and transcript levels decrease significantly as cells enter stationary phase. The genes encoding tellurite resistance are functional in EHEC, and can be separated into two classes – those that are expressed constitutively during growth (*terDEZ*) and those that are expressed only during growth in the presence of tellurite (*terBCF) *[96]. Our data show this as well, with *terD, E*, and *Z *all expressed during exponential growth, with a significant decrease in transcript levels upon entry to stationary phase, and no significant changes in transcript levels for *terA *and *F*.

As *E. coli *O157:H7 cells transitioned to stationary phase, we observed a transient increase in expression of ORFs encoding proteins involved in acquisition and utilization of alternative carbon sources. ORFs involved in acetate uptake and utilization increased in expression, and some of these genes, such as *acs*, are known to be controlled, in part, by RpoS [46]. RpoS is activated during slow growth on glucose [52], and growth on less desirable carbon sources [100]. RpoS is also activated during the switch from one carbon source to another, as observed in *E. coli *K-12 growing in a rich medium [33] and in minimal medium [23]. The glyoxalate shunt is thought to play an important role under glucose-limited conditions, where the absence of catabolite repression is an important factor for activity of the cycle [101]; here we observed that ORFs encoding glyoxalate shunt enzymes increased in expression as *E. coli *O157:H7 entered stationary phase (Tables 3 and 4). Over expression of high affinity sugar transporters occurs during the short-term adaptation of *E. coli *K-12 to carbon-limited growth [52,102]. Sugar transporters and carbon uptake systems are also activated in *E. coli *K-12 during growth on less desirable carbon sources [100]. In *E. coli *O157:H7, we observed increased expression of ORFs encoding transport systems for carbohydrates, sugar alcohols and acids during the transition to stationary phase. These data suggest that as glucose was depleted, other carbon sources were sought, but were not available in the minimal medium. In a rich medium, we would expect cells to resume growth, albeit at a lower rate, once glucose was consumed.

Transition to stationary phase is characterized by complex physiological changes to the bacterial cell. Many of the changes in expression patterns observed for O157:H7 in this study have also been observed for *E. coli *K-12 entering stationary phase [23]. Growth under stressful conditions, such as in the presence of acetate [79] or at low pH under anaerobic conditions [28], also induce expression of stationary phase associated genes. The sigma factor RpoS plays a critical role in transcribing genes associated with stationary phase and stress response [24-26], many of these genes, such as *bolA, dksA, osmC*, and *osmB*, have been identified in this study as well. We still do not know about most of the O157-specific ORFs, and what regulators control their expression. Here we have classified some of the well-known O157 genes, as well as O157 genes of unknown function, as being modulated during growth transitions. Global transcriptional profiling of isogenic mutants for a specific regulator have been conducted for a number of regulators in *E. coli *K-12. As we know more about the regulation of virulence loci, such as the LEE, by global regulators, it would be very useful to perform these profiling experiments in *E. coli *O157:H7 and other pathogenic *E. coli*.

## Conclusion

The principal contribution of this study is the first complete description of global transcriptome profiling of the human pathogenic strain, *E. coli *O157:H7, during growth transitions. Many O157-specific ORFs responded to the growth transition with significant changes in gene expression including multiple genes that are encoded on pathogenicity islands or toxin-converting bacteriophages. Because a majority of the O157-specific ORFs have unknown functions, these findings will have practical use for comparing transcriptional responses to a variety of growth conditions and elucidating the function and factors regulating these unknown ORFs, some of which may contribute to disease processes. These results also generate new hypotheses and provide opportunities to identify ORFs of interest, based on expression profiles, and compare expression within *E. coli *O157:H7 populations or with other EHEC populations. It is useful to understand what transcriptional changes occur from exponential to stationary phase, as these transitions are important adaptations to the growth and survival of *E. coli *O157:H7 in natural conditions.

## Methods

### Growth conditions

*E. coli *O157:H7 RIMD0509952 (Sakai) involved in a radish sprout outbreak [103] was stored at -70°C in LB broth and 10% glycerol. The Sakai strain was inoculated into 10 mL of LB broth from freezer stocks and grown to OD_600 _= 0.1. This ~4 h period of growth in LB allowed cells to recover before transfer to MOPS minimal media. MOPS minimal media (10×) was prepared as described in Neidhardt et al. [104] with the addition of 20 mL of micronutrient solution instead of 10 mL. MOPS minimal media contained 100 mL of 10× MOPS minimal media, 10 mL of 0.132 M K_2_HPO_4_, and 5 mL of 20% D-glucose per liter. Cultures were inoculated into 50 mL of MOPS minimal media with 0.1% glucose at a ratio of 1:200 and grown to stationary phase at 37°C with shaking. Cultures in MOPS were transferred to 100 mL MOPS in 250 mL plastic culture flasks (Nalgene, Rochester, N.Y.) at a ratio of 1:75 and grown for 9 h before transfer again to 100 mL MOPS in 250 mL plastic culture flasks at a ratio of 1:30 and this culture was used to sample RNA during growth (Fig. 1). Four independent cultures were sampled at 3 h (OD_600 _≈ 0.22), 4 h (OD_600 _≈ 0.45), 4.33 h (OD_600 _≈ 0.73), 4.66 h (OD_600 _≈ 0.93), 5 h (OD_600 _≈ 1), 5.5 h, 6 h, 7 h, 7.5 h, and 8 h. After 5 h, the culture density remained at OD_600 _≈ 1. Residual dissolved oxygen tension was monitored using a dO_2 _probe and Consort C535 multimeter (Topac Instrumentation, Hingham, Mass.) for three independent cultures in MOPS minimal medium.

### RNA isolation

At each time point, 4 mL of culture was mixed with 8 mL of RNAProtect (Qiagen, Valencia, Calif.), vortexed, and centrifuged at 4°C, 7500 rpm for 10 min to pellet cells. The supernatant was removed and cell pellets stored at -70°C for 1 week or less before RNA extraction. Cell pellets were suspended in 700 uL of 95C lysis buffer (20 mM sodium acetate pH 5.2, 2 mM EDTA pH 8.0, and 0.5% SDS), held at 95°C for 30s then mixed with 700 uL of 65°C Acid-Phenol: Chloroform, pH 4.5 (with IAA, 125:25:1) (Ambion, Austin, Tex.). Samples were held at 65°C with periodic shaking for at least 6 minutes before centrifuging at 12000 rpm for 10 min. Supernatant was extracted again with acid-phenol:chloroform and then with chloroform:isoamyl alcohol (24.1). RNA was precipitated for at least 1 hour at -20°C in 2.5 V 100% ethanol and 1/10 V 3 M sodium acetate pH 5.2. RNA was suspended in 1 mM sodium citrate pH 6.5 and stored at -70°C. RNA samples were purified and treated with Dnase using the Rneasy kit (Qiagen). RNA quality was assessed by electrophoresis on formaldehyde-agarose gel. RNA was checked for complete DNA digestion by PCR.

### cDNA synthesis and hybridizations

Reverse transcription reactions contained 6 ug RNA, 2 ug random primers (Invitrogen, Carlsbad, Calif.), 1× first strand buffer (Invitrogen), 10 mM DTT, 400 U Superscript II (Invitrogen), 0.5 mM each dATP, dCTP, and dGTP, 0.3 mM dTTP, and 0.2 mM amino-allyl dUTP. 30 uL reactions were incubated overnight at 42°C. cDNA was purified using PCR cleanup columns (Qiagen) with Phosphate wash buffer (5 mM K_2_HPO4, pH 8.0, 80%EtOH) and phosphate elution buffer (4 mM K_2_HPO4, pH 8.5). Amino-allyl labeled cDNA was dried and suspended in 0.1 M sodium carbonate pH 9.3 and coupled with either Cy3 or Cy5 (Amersham Biosciences, Piscataway, N.J.). Uncoupled dye was removed by another purfication using the PCR cleanup kit. Concentration of cDNA and amount of incoporated dye was measured for each sample using a Nanodrop spectrophotometer (Ambion).

### Oligo microarray

The *E. coli *oligo set version 1.0.1 (Operon) was printed onto Corning UltraGaps (Corning Incorporated, Acton, Mass.) coated slides at the Research Technology Support Facility at Michigan State University. The Qiagen oligo set contained 5,978 probes specific for three *E. coli *strains, K-12 (MG1655) [105], O157:H7 Sakai [16], and O157:H7 EDL-933 [17], in which 5,943 probes were 70-mer oligonucleotides, and 35 probes had lengths that ranged from 41–69 bp. All of these probes are spotted in duplicate on each array. The oligo set also contained 12 randomized negative control 70-mer oligonucleotides. There are a common set of 3807 probes that target 3807 ORFs present based on the genome sequences of *E. coli *K-12 MG1655, *E. coli *O157:H7 EDL-933, and O157:H7 Sakai, which we refer to as the backbone ORFs. There are also 1741 probes targeting ORFs that are specific to one or two of the genomes, typically to O157:H7 EDL-933 and Sakai. These probes are referred to as the O157-specific ORFs. Probes designed to target K-12-specific or EDL-933 specific ORFs were not considered after the data analysis stage because these genes are not present in the Sakai genome. We used the Qiagen annotation for the probe set, and analyzed only the 4886 probes that were assigned a ECs number.

### Hybridization conditions

Arrays were cross-linked by exposure to 600 mJ UV before blocking in 1% SDS, 5× SSC, and 1 mg/mL BSA at 42°C for 1 hour. After blocking, arrays were washed 2× 5 min in 0.1× SSC and 2× 30 s in H2O. Dried arrays were placed into hybridization cassettes (TeleChem International, Sunnyvale, Calif.) and the cDNA samples were suspended in 10 mM EDTA, denatured at 95°C for 5 min and then mixed with 40 uL of SlideHyb buffer 1 (Ambion) and loaded under a coverslip onto the array. Hybridizations were carried out at 47°C for 16–18 h. After hybridization, arrays were washed in 2× SSC, 0.5% SDS 37C for 5 min, followed by 2× 5 min in 0.1× SSC, 0.1% SDS 37°C, and then 2 × 2.5 min in room temperature 0.1× SSC. Arrays were scanned using an Axon 4000b scanner (Molecular Devices, Sunnyvale, Calif) and images were analyzed using GenePix 6.0 (Molecular Devices). The 3 h sample served as a common reference and was hybridized with all subsequent samples. Array data have been deposited at the NCBI Gene Expression Omnibus (Accession GSE7477).

### Quantitative real-time PCR

Expression levels of 14 ORFs determined to be differentially expressed (p < 0.00001) were verified by quantitative real-time PCR (Q-PCR). Primer pairs were designed based on the published reference genome sequence of *E. coli *O157:H7 strain Sakai using the Primer3 server, and secondary structure scrutinized using the Mfold algorithm [106,107] (Additional File 4). cDNA was synthesized from 1 ug of total RNA using the iScript Select cDNA synthesis kit (BioRad, Hercules, Calif.) and random hexamers supplied with the kit. Template cDNA was diluted to 10^-1 ^to 10^-3 ^for use in Q-PCR. Q-PCR reactions contained 12.5 uL 2× iQ SYBR green supermix (BioRad), 0.63 uL of each primer (10 uM stock), 9.24 uL H2O, and 2 uL cDNA and were conducted under the following conditions: 2 min at 95C, followed by 40 cycles of 10 sec at 95C then 20 sec at the specific annealing temperature. Q-PCR reactions were performed in triplicate for each cDNA sample tested. Relative expression was determined using the method described by Pfaffl et al. [108] and the fluorescence data from the 16S rRNA target were used for normalization within samples. All samples were then compared to the expression levels of the mid-exponential (3 hour) samples. The average log2 expression and standard deviation from two independent RNA samples are reported for each time point tested.

### Data analysis

Raw intensity values for all probes on each array were normalized using pin-tip LOWESS [109] in R v.2.2.1 [110] with the MAANOVA (v. 0.98–8) package [111]. Signals from two replicate probes on each array were averaged and log_2 _transformation applied. Differences in transcription levels over time were determined using a mixed model ANOVA in R/MAANOVA, where the log transformed intensity data, Y = A (array) + D (dye) + T (time) + S (sample = biological replicate) + E (error). The ANOVA modeling allows for consideration of appropriate error structures for experiments with multiple sources of variation in microarray measurements [112]. The random effects of the model were biological replicate and array effects, whereas the fixed effects were time point and dye effects [113]. The Fs statistic, a shrinkage estimator for gene-specific variance components that makes no assumptions about the distribution of variances across genes, was estimated [40]. Significant changes in expression over time were determined by calculating the p values for the Fs statistic for each gene using 1000 random permutations. The p-values were adjusted to correct for type I error with the Benjamini-Hochberg (B-H) linear step-up correction implemented in R/MAANOVA and a cutoff adjusted p-value of 0.0000001. Pair wise contrasts of time points were estimated by the t-test in R/MAANOVA. Contrast p-values were corrected for multiple testing by using the B-H step-up correction. Probes with significant changes in expression over time were grouped by the time points at which the significant change occurred, as determined by the contrast analysis (adjusted p value < 0.05). QT clustering of significant ORFs was conducted in MeV v. 3.1 [116] with a diameter of 0.4 and a minimum cluster size of 15.

Gene Set Enrichment Analysis (GSEA) [114] was conducted using GSEA v. 2.0 [115] on a ranked list of log_2 _expression ratios (determined from the ANOVA estimates) for 5 of the time points relative to the mid-exponential time point. The following parameters were used for the analysis: 1000 permutations and exclusion of gene sets with less than 5 members and greater than 700 members. Gene sets were defined based on the *E. coli *O157:H7 Sakai role categories obtained from the Comprehensive Microbial Resource at The Institute for Genomic Research [116]. The GSEA results are summarized in Additional File 4. All gene sets determined to be enriched with a FDR (q-value) less than 0.25 are reported.

## Authors' contributions

TMB designed and carried out all the microarray experiments, data analysis, and composed the manuscript. LMW and WQ assisted in the experimental design and statistical data analyses. JTR designed and optimized the Q-PCR assays and LMO conducted the experimental assays. TSW oversaw the project, contributed to the design and final analyses, and manuscript completion. All authors have read and approved the final version of the manuscript.

## Supplementary Material

Additional File 1 Entire data set of O157 ORFs sorted by significance. Log2 expression indices for each time point for 4886 ORFs in the multigenome microarray Click here for file

Additional File 2 Significant O157-specific ORFs. Log2 expression indices for each time point Click here for file

Additional File 4 GSEA results. Gene Set Enrichment Analysis (GSEA) conducted using GSEA v. 2.0 on a ranked list of log_2 _expression ratios (determined from the ANOVA estimates) for 5 of the time points relative to the mid-exponential time point. Click here for file

Additional File 3 QPCR primers. PCR primers designed for QPCR analysis. Click here for file
