# Supplementary material for: Global transcriptional response of Escherichia coli O157:H7 to growth transitions in glucose minimal medium
Source: BMC Microbiol. 2007 Oct 29;7:97. doi: 10.1186/1471-2180-7-97 (PMC2241611; doi:10.1186/1471-2180-7-97)
Supplement: Additional File 3 — QPCR primers. PCR primers designed for QPCR analysis. [file 1471-2180-7-97-S3.doc]

**Supplemental Table 3.** Primer sequences used for quantitative PCR.

| Primer | Sequence (5’ to 3’) | Annealing temperature |
| --- | --- | --- |
| espA-128 | AGGCTGCGATTCTCATGTTT | 57ºC |
| espA-310 | GAAGTTTGGCTTTCGCATTC |
| espB-319 | TCAGCATTGGGGATCTTAGG | 57ºC |
| espB-487 | CTGCGACATCAGCAACACTT |  |
| espZ-46 | GCGACCTCACTCAGTGGAA | 55ºC |
| espZ-193 | CCGCTGCAATACCTGTACCT |  |
| eae-2188 | GCCGGTAAAGCGACTGTTAG | 55ºC |
| eae-2325 | ATTAGGCAACTCGCCTCTGA |  |
| tir-664 | ACTTCCAGCCTTCGTTCAGA | 57ºC |
| tir-869 | TTCTGGAACGCTTCTTTCGT |  |
| espJ-512 | CAGCTTCTGTCGGCTCTCTT | 55ºC |
| espJ-611 | TCTGGCAGCGTAAAGATGAA |  |
| espY1-61 | TATAAGATGGTGAGCGGGGA | 55ºC |
| espY1-164 | GCCTCAGTCTGTTTATCTGACCA |  |
| stx1A-524 | CTGTGGCAAGAGCGATGTTA | 57ºC |
| stx1A-686 | CTCAACCTTCCCCAGTTCAA |  |
| terZ-239 | ACAGTGGCGATAACCTGACC | 57ºC |
| terZ-432 | CTTGTAGCGAGCCAGCTCTT |  |
| ureD-237 | TCTCCAGACGGTTAGCGAGT | 55ºC |
| ureD-361 | CACGTTGCATACCGTGTTTC |  |
| espM1-420 | AATGGATGCTCTTTGCGATGGTA | 57ºC |
| espM1-526 | CCGGGGCGATATGTTTCTGAT |  |
| rrs-982 | CGATGCAACGCGAAGAACCT | 55ºC |
| rrs-1143 | CCGGACCGCTGGCAACAAA |  |
| stx2A-357 | TATATCAGTGCCCGGTGTGA | 55ºC |
| stx2A-464 | TGACGACTGATTTGCATTCC |  |
| stx2B-57 | GGCGGATTGTGCTAAAGGTA | 55ºC |
| stx2B-185 | TGAGCACTTTGCAGTAACGG |  |
